# Synergistic performance of a new bimetallic complex supported on magnetic nanoparticles for Sonogashira and C–N coupling reactions

**DOI:** 10.1038/s41598-023-44168-6

**Published:** 2023-10-24

**Authors:** Fatemeh Nasseri, Mohammad Ali Nasseri, Mohamad Zaman Kassaee, Issa Yavari

**Affiliations:** 1 Department of Chemistry, Tarbiat Modares University, P.O. Box 14155-175, Tehran, Iran; 2 Department of Chemistry, Faculty of Basic Sciences, University of Birjand, P.O. Box 97175-615, Birjand, Iran

**Keywords:** Organic chemistry, Natural product synthesis, Reaction mechanisms, Catalysis, Catalyst synthesis, Heterogeneous catalysis

## Abstract

This paper describes the synthesis of a novel Cu–Ni bimetallic system comprising of magnetic nanoparticles, as the core, and 4-amino-3,5-bis(pyridin-2-yl)-1,2,4-triazole (4-ABPT), as a conjugated bridge, between nickel and copper species. With low Cu and Ni loading (0.06 mol% Ni, 0.08 mol% Cu), the resulting Fe_3_O_4_@SiO_2_@4-ABPT/Cu–Ni showed to be a highly efficient catalyst for the Sonogashira and C–N cross-coupling reactions. The developed catalyst was well characterized by FT-IR, XRD, EDX-mapping, FE-SEM, TEM, ICP, VSM, TGA/DTG/DTA, LSV, and XPS techniques. Fe_3_O_4_@SiO_2_@4-ABPT/Cu–Ni nanocatalyst was compatible with a wide range of amines and aryl halides in the Sonogashira and C–N cross-coupling reactions and offered desired coupling products in high to excellent yields under palladium- and solvent-free conditions. Based on the XPS results, the 4-ABPT ligand can adjust electron transfer between Ni and Cu in Fe_3_O_4_@SiO_2_@4-ABPT/Cu–Ni, promoting the formation and stabilization of Cu^+^ and Ni^3+^ species. Electronic interactions and the synergistic effect between these metals increased the selectivity and activity of Fe_3_O_4_@SiO_2_@4-ABPT/Cu–Ni catalyst in the Sonogashira and C–N cross-coupling reactions compared with its monometallic counterparts. Additionally, the magnetic properties of Fe_3_O_4_@SiO_2_@4-ABPT/Cu–Ni facilitated its separation from the reaction mixture, promoting its reuse for several times with no significant loss in its catalytic activity or performance.

## Introduction

Carbon–carbon and carbon-heteroatom cross-coupling reactions, which traditionally apply palladium catalysts, are among the most significant chemical processes in organic synthesis^1–5^. A look at the development of these reactions indicates their applications in materials science, agrochemical compounds, electronic materials, and polymers area^6–8^. For almost two decades, they have significantly influenced drug discovery and medicinal chemistry^9–11^.

Despite the considerable prospects of palladium catalysts in cross-coupling reactions, the high cost and possible toxicity of Pd have remained a scientific challenge, highlighting the need for developing and discovering alternative ways with an environmentally friendly and non-toxic catalytic system^12–14^. Numerous studies have reported the use of other transition metals, such as Ni^15,16^, Cu^17,18^, Fe^19,20^, and Co^21,22^ for more appropriate, cost-effective, and safe approaches for cross-coupling reactions, over the past decade. Considerable attention has been given to copper catalysis, owing to its good functional group tolerance, the low cost of the catalysts, and low toxicity^23,24^. Besides, copper plays an essential role in cross-coupling reactions. Its scope and function in the bond-formation processes of C–heteroatom and C–C bonds have considerably increased^11,25^.

Copper is crucial for cross-coupling reactions, but it suffers from several drawbacks like low activity and a propensity for oxidative homo-coupling reactions^21,26^. The drawbacks can be optimized by the introduction of a second metal into a Cu monometallic sample as a Cu–M bimetallic catalytic system. As a result, the geometric and electronic properties of the sample can be modified^27,28^. The synergistic effect of both metals improves the efficiency and selectivity of the catalyst^11,29^.

Nickel is an ideal candidate as the second metal for the following reasons. Ni has a high bonding affinity with sufficient flexibility to generate multiple oxidation states^30,31^. Furthermore, metal-catalyzed cross-coupling processes show that nickel is as reactive as palladium^26^.

Over the past 20 years, several attempts have been made to develop heterogenous Cu–Ni bimetallic catalysts for improved cross-coupling reactions. In 2008, Lipshutz et al. synthesized a heterogeneous bimetallic catalyst of copper and nickel oxide particles supported within charcoal (Ni/Cu@C). It was the first example of a mixed-metal, recyclable catalyst composed of Cu and Ni that could mediate both groups 10 and group 11 cross-couplings^32^. Varadwaj and co-workers, in 2013, studied an amine-functionalized montmorillonite-supported Cu, Ni catalyst for C–S coupling reactions. As a bimetallic catalytic system, the strong synergistic interaction of Cu and Ni increased the yield of C–S couplings^27^. Recently, Nasresfahani et al. reported Ni/Cu-MCM-41 as a reusable and efficient bimetallic catalyst for the Sonogashira cross-coupling reactions^33^.

Filtration or centrifugation techniques can be utilized to recover the majority of these heterogeneous catalysts. However, these processes are time-consuming and contaminate the product as the catalyst particles are lost. This issue can be resolved by employing magnetic Fe_3_O_4_@SiO_2_ nanoparticles as a heterogeneous recoverable solid support with a high surface area. Conventional magnets can be simply used to separate the magnetically immobilized catalyst from the reaction media.

The existence of organic ligands on the support surface can facilitate the anchoring of metal ions/metals via chelation^34,35^. The properties of metal-complexed ligands may also regulate the activity and selectivity of the catalyst^36,37^. In bimetallic complexes, bridging ligands can be crucial in metal–metal interactions^38–42^. According to the through-bond super-exchange formalism, bridging ligands with π-conjugated structures are favored for accelerating electron transfer^43^. One of the most popular chelating ligands, which can be used as a bridge between metallic centers, is 4-Amino-3,5-bis(pyridin-2-yl)-1,2,4-triazole (4-ABPT)^44^.

**Figure 1:**
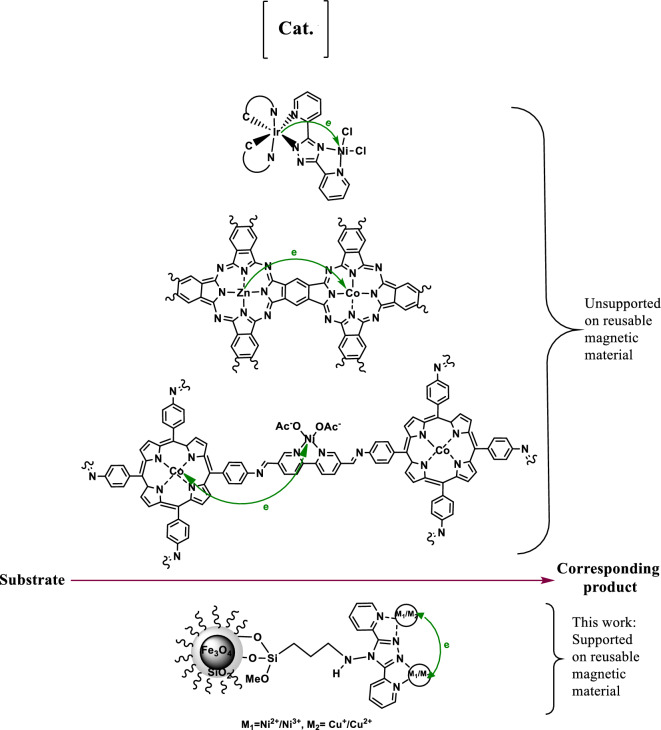
Importance of the π-conjugated bimetallic systems as the catalytic systems for chemical reactions.

In this work, 4-ABPT was utilized as a ligand with a large conjugated system to synthesize Fe_3_O_4_@SiO_2_@4-ABPT, as a suitable support, for stabilizing bimetallic Cu–Ni nanoparticles. To the best of our knowledge, there are some π-conjugated bimetallic systems that were unsupported on reusable magnetic materials and used as the catalytic systems for chemical reactions ^40,45,46^, but Fe_3_O_4_@SiO_2_@4-ABPT/Cu–Ni is the first example of a π-conjugated system between two metals supported on reusable magnetic materials (Fig. 1). The resultant Fe_3_O_4_@SiO_2_@4-ABPT/Cu–Ni nanocatalyst had excellent catalytic activity for Sonogashira and C–N cross-coupling reactions under solvent-free conditions. The present Cu–Ni bimetallic system benefits from the cooperativity between conjugated 4-ABPT ligand and Cu/Ni centers. Moreover, the Fe_3_O_4_ magnetic core in Fe_3_O_4_@SiO_2_@4-ABPT/Cu–Ni nanocatalyst provides appropriate reusability in the cross-coupling reactions.

## Experimental section

### Materials and methods

All chemicals were purchased from Merck Company with high purity. NMR spectra were recorded using a Bruker Avance DPX-250 (^1^H-NMR at 250 MHz and ^13^C–NMR at 62.5 MHz) spectrometer in the deuterated (CDCl_3_ and DMSO-d_6_) solvents and TMS as the internal standard. The purity of the products and the reaction progress were evaluated by thin layer chromatography (TLC) on silica-gel Polygram SILG/UV254 plates. Fourier transform infrared (FT-IR) spectra were recorded on a JASCO FT/IR 4600 spectrophotometer using a KBr pellet. Melting points were measured on an Electro thermal 9100 apparatus. The images of field-emission scanning electron microscopy (FE-SEM) were taken by a Tescan Mira3 microscope. Energy-dispersive X-ray (EDX) spectroscopy was performed using a scanning electron microscope (SEM, FEI Quanta 200) equipped with an EDX detector. The presence of the elements was confirmed using the point elemental mapping (TESCAN MIRA 3 LMU). Transmission electron microscopy (TEM) analysis was accomplished by a TEM microscope (Philips EM 208S) operating at 100 kV. Thermogravimetry and differential thermal analysis (TGA-DTA) were performed using a Q600 model from TA Company; the sample was heated from 25 to 1000 °C at the rate of 10 °C min^−1^ under a nitrogen atmosphere. Vibrating-sample magnetometer (VSM) analysis was performed by LBKFB model-magnetic Kashan kavir. X-ray diffraction (XRD) patterns were obtained by a Philips-PW 1730 X-ray diffractometer incorporating Cu Kα radiation (λ = 0.154 nm). X-ray photoelectron spectroscopy (XPS) analyses were implemented using a Thermo Scientific K-Alpha XPS system (Thermo Fisher Scientific, U.S.A). The XPS spectra were deconvoluted by using Gaussian–Lorentzian curves. Linear sweep voltammetry (LSV) (Electrochemical measurements) of the samples was assessed by a three-electrode system including carbon paste electrode modified (MCPE) with Fe_3_O_4_@SiO_2_@4-ABPT/Cu, Fe_3_O_4_@SiO_2_@4-ABPT/Ni, and Fe_3_O_4_@SiO_2_@4-ABPT/Cu–Ni as working electrodes, saturated calomel electrode (SCE) as reference electrode and platinum wire as an auxiliary electrode (Azar Electrode Co, Iran), using Ivium galvanostat/potentiostat (CmpactStat, Switzerland). Finally, the Cu and Ni contents on the catalyst were analyzed by an inductively coupled plasma-optical emission spectrophotometer (ICP-OES, 730-ES, Varian Inc.).

### Synthesis of magnetic iron oxide (Fe_3_O_4_) nanoparticles

The Fe_3_O_4_ nanoparticles were synthesized via chemical co-precipitation using chlorine salts of Fe^3+^ and Fe^2+^ ions with a molar ratio of 2:1 in the presence of an ammonia solution, followed by the hydrothermal treatment. Typically, a mixture of FeCl_3_·6H_2_O (1.76 g, 6.5 mmol) and FeCl_2_.4H_2_O (0.65 g, 3.3 mmol) was dissolved in the deionized water (100 mL), and the solution was vigorously stirred for 1 h under an argon atmosphere. Afterward, NH_4_OH (6 mL of 25%) was dropwise added to the reaction mixture. The mixture was heated for 1 h at 80 °C, and the cooled black magnetite solid was collected with an external magnet, washed with distilled water, and dried under vacuum at 50 °C for 24 h.

### Synthesis of silica-coated magnetic nanoparticles (Fe_3_O_4_@SiO_2_)

Fe_3_O_4_ NPs (1 g) were dispersed in ethanol and distilled water (40:10 mL) under ultrasonication for 30 min. The pH was adjusted to 10 with an ammonia solution and then 1 mL tetraethyl orthosilicate (TEOS) was slowly dropped into the mixture over 10 min. After mechanical stirring for 12 h at 60 °C, the obtained Fe_3_O_4_@SiO_2_ nanoparticles were separated by an external magnet, washed several times with ethanol, and dried under vacuum.

### Synthesis of chloro-functionalized silica-coated magnetite nanoparticles (Fe_3_O_4_@SiO_2_–Cl)

A mixture of Fe_3_O_4_@ SiO_2_ (1.0 g) was dispersed in dry toluene (30 mL) by sonication for 45 min. Next, 3-chloropropyl trimethoxysilane (CPTMS, 1 mL) was dropwise added to the mixture and slowly heated to 105 °C. The mixture was stirred using a mechanical stirrer for 20 h under an argon atmosphere. After cooling to room temperature, the resulting chloro-functionalized Fe_3_O_4_@SiO_2_ was collected using an external magnet, washed several times with CH_2_Cl_2_ and Et_2_O, and dried under vacuum^47,48^. The Cl atom loading was 0.42 mmol per gram of catalyst based on the elemental analysis.

### Synthesis of 4-Amino-3,5-bis(pyridin-2-yl)-1,2,4-triazole supported on Fe_3_O_4_@SiO_2_ nanoparticles (Fe_3_O_4_@SiO_2_@4-ABPT)

4-Amino-3,5-bis(pyridin-2-yl)-1,2,4-triazole (4-ABPT) was synthesized according to the literature procedure^49^. For the preparation of the supported 4-ABPT ligand, Fe_3_O_4_@SiO_2_–Cl (0.3 g), 4-Amino-3,5-bis(pyridin-2-yl)-1,2,4-triazole (0.3 g, 1 mmol) and triethylamine (14 mL, 0.1 mol) were refluxed in dry toluene (40 mL) at 100 °C for 24 h under argon atmosphere using a round-bottom flask fitted with a mechanical stirrer and condenser. Then, the obtained product (Fe_3_O_4_@SiO_2_@4-ABPT) was separated with a permanent magnet, washed several times with toluene, and dried under vacuum.

### Synthesis of Fe_3_O_4_@SiO_2_@4-ABPT anchored Cu–Ni nanoparticles (Fe_3_O_4_@SiO_2_@4-ABPT/Cu–Ni)

Finally, for the preparation of the copper-nickel bimetallic catalyst (Fe_3_O_4_@SiO_2_@4-ABPT/Cu–Ni), 0.4 g of Fe_3_O_4_@SiO_2_@4-ABPT was dispersed with Cu(OAc)_2_·H_2_O (0.02 g) and Ni(OAc)_2_.4H_2_O (0.02 g) in ethanol (60 mL) to achieve 5 wt% of Cu and 5 wt% of Ni. The mixture was vigorously stirred for 12 h at 80 °C. Then, the cooled solid was collected with an external magnet and washed with EtOH (3 × 10 mL) .

### General procedure for the Sonogashira cross-coupling reaction catalyzed by Fe_3_O_4_@SiO_2_@4-ABPT/Cu–Ni

A mixture of phenylacetylene (1.5 mmol), aryl halide (1.0 mmol), NaO^*t*^Bu (1.0 mmol), and Fe_3_O_4_@SiO_2_@4-ABPT/Cu–Ni catalyst (0.01 g, 0.06 mol% Ni, 0.08 mol% Cu) was stirred at 120 °C under solvent-free conditions. TLC was utilized to monitor the progress of the reaction. After completion of the reaction, the reaction mixture was diluted with EtOAc (5 mL). Using an external magnet, Fe_3_O_4_@SiO_2_@4-ABPT/Cu–Ni was separated as the catalyst and washed with EtOAc (2 × 10 mL) and EtOH (2 × 10 mL), dried under vacuum, and reused. Pure products were obtained by column chromatography (silica gel) using a 4:1 volume ratio of *n*-hexane: EtOAc as eluent.

### General procedure for C–N cross-coupling reaction catalyzed by Fe_3_O_4_@SiO_2_@4-ABPT/Cu–Ni

A mixture of aryl halide (or phenylboronic acid, 1.0 mmol), *N*-heterocyclic compound (1.3 mmol), NaO^*t*^Bu (1.0 mmol), and Fe_3_O_4_@SiO_2_@4-ABPT/Cu–Ni catalyst (0.01 g, 0.06 mol% Ni, 0.08 mol% Cu) was stirred at 120 °C under solvent-free conditions for the desired reaction time under TLC monitoring. Further, the reaction mixture was extracted with ethyl acetate (5 mL). The catalyst was separated with an external magnet, washed with EtOAc (2 × 10 mL) and EtOH (2 × 10 mL), and dried in vacuum. The pure coupling product was obtained by column chromatography (silica gel) using a 10:2 volume ratio of *n*-hexane: EtOAc as eluent.

## Results and discussion

**Figure 2:**
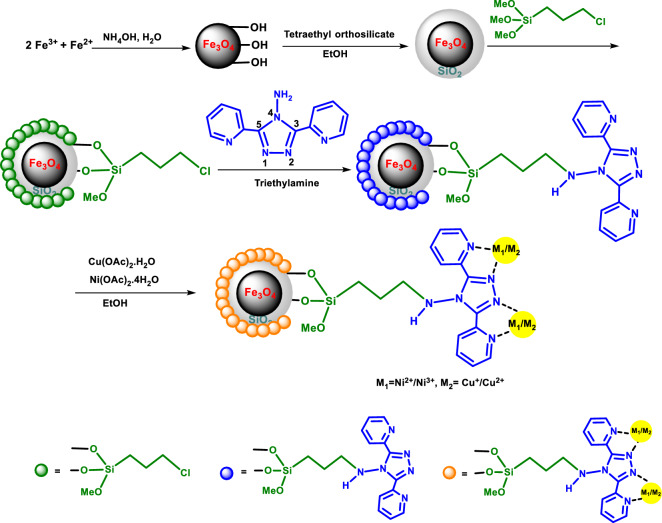
Preparation of Fe_3_O_4_@SiO_2_@4-ABPT/Cu–Ni nanocatalyst.

Figure 2 illustrates the approach used to prepare a Cu–Ni bimetallic catalyst (Fe_3_O_4_@SiO_2_@4-ABPT/Cu–Ni). Initially, Fe_3_O_4_ NPs were synthesized through the chemical co-precipitation method, followed by silica coating by TEOS. Then, Fe_3_O_4_@SiO_2_ was functionalized through reacting with 3-chloropropyl trimethoxysilane and subsequent treatment with 4-Amino-3, 5-bis(pyridin-2-yl)-1,2,4-triazole to produce Fe_3_O_4_@SiO_2_@4-ABPT. Finally, Cu–Ni nanoparticles were immobilized onto Fe_3_O_4_@SiO_2_@4-ABPT by adding Cu(OAc)_2_·H_2_O and Ni(OAc)_2_·4H_2_O precursors. The formation of the bimetallic system was verified using FT-IR spectroscopy, XRD, VSM, TGA, FE-SEM, EDX-mapping, TEM, XPS, and ICP techniques.

**Figure 3:**
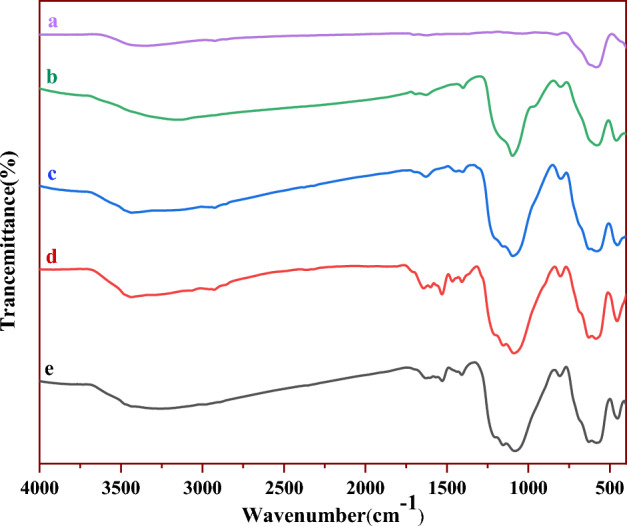
FT-IR spectra of (**a**) Fe_3_O_4_, (**b**) Fe_3_O_4_@SiO_2_, (**c**) Fe_3_O_4_@SiO_2_@CPTMS, (**d**) Fe_3_O_4_@SiO_2_@4-ABPT, and (**e**) Fe_3_O_4_@SiO_2_@4-ABPT/Cu–Ni.

FT-IR spectra of Fe_3_O_4_, Fe_3_O_4_@SiO_2_, Fe_3_O_4_@SiO_2_@CPTMS, Fe_3_O_4_@SiO_2_@4-ABPT, and Fe_3_O_4_@SiO_2_@4-ABPT/Cu–Ni are depicted in Fig. 3. Curve **a** exhibits a strong absorption band at around 591 cm^−1^ which can be attributed to the Fe − O stretching vibrations in Fe_3_O_4_^50^. A broad high-intensity band near 1100 cm^-1^ in curve **b** can be ascribed to the Si–O-Si asymmetric stretching vibrations, while the weaker band at 800 cm^−1^ shows the symmetric stretching vibration of the Si–O–Si^51,52^. These results indicate that the silica layer is well formed around the magnetic core. Curve **c** exhibits the anchor of CPTMS onto the surface of Fe_3_O_4_@SiO_2_ NPs by C–H stretching vibrations that appeared at ~ 2928 cm^−1^^53,54^. Curve **d** demonstrates a peak at ~ 1652 cm^−1^ due to the stretching vibration of C═N in the pyridine ring. Also, the peak at about 1531 cm^−1^ can be ascribed to the N–H bending vibration. Furthermore, the IR spectrum illustrates two bands near 1468 and 1600 cm^−1^ corresponding to the aromatic rings. These vibrational bands suggest that the surface of Fe_3_O_4_@SiO_2_ nanoparticles is successfully modified with the 4-ABPT ligand. Curve **e** shows a slight shift and variation in the amplitude of the N–H (a shift from 1531 to 1526 cm^−1^) and C=N (a shift from1652 to 1640 cm^−1^) bands of triazole, which can verify the coordination between the metals and the nitrogen atoms of Fe_3_O_4_@SiO_2_@4-ABPT/Cu–Ni structure.

**Figure 4:**
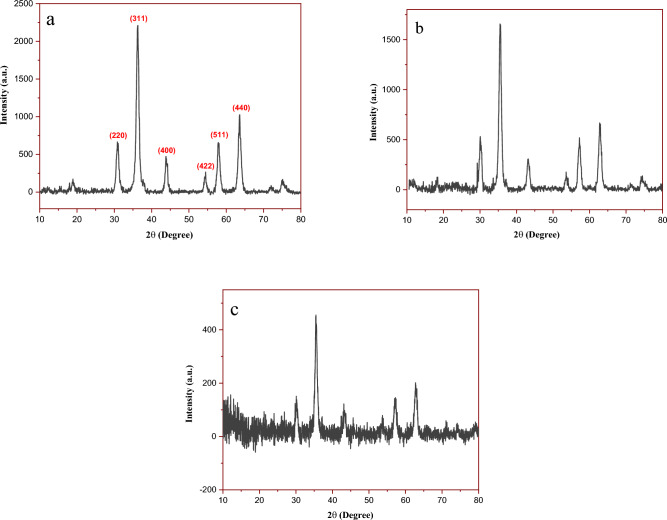
XRD patterns of (**a**) Fe_3_O_4_, (**b**) Fe_3_O_4_@SiO_2_ and (**c**) Fe_3_O_4_@SiO_2_@4-ABPT/Cu–Ni.

Figure 4 shows the XRD patterns of Fe_3_O_4_, Fe_3_O_4_@SiO_2_, and Fe_3_O_4_@SiO_2_@4-ABPT/Cu–Ni nanoparticles. The XRD pattern of Fe_3_O_4_, shows six main characteristic peaks at 2 θ**  = **30.31°, 35.91°, 43.87°, 54.01°, 57.66°, and 63.57°, corresponding to (220), (311), (400), (422), (511), and (440) planes, respectively (Fig. 4a). These reflections firmly verified the crystal structure of Fe_3_O_4_ consistent with that reported in the literature (JCPDS card no. 19-629)^55,56^. The same sets of characteristic peaks were also observed in the case of Fe_3_O_4_@SiO_2_ NPs, suggesting the presence of the crystalline Fe_3_O_4_ NPs in their structures (Fig. 4b). Due to the amorphous structure of silica-coated on the Fe_3_O_4_ NPs, the SiO_2_ peak was observed at 2θ = 15–25° in the XRD patterns of Fe_3_O_4_@SiO_2_^57^. The presence of peaks corresponding to the Fe_3_O_4_ structure, as well as the amorphous silica peak, in the XRD pattern of Fe_3_O_4_@SiO_2_@4-ABPT/Cu–Ni (Fig. 4c) indicates that the surface modification of the Fe_3_O_4_ NPs caused no change in their stability and crystalline structure. Moreover, a noticeable reduction can be seen in the intensity of the peaks of Fe_3_O_4_@SiO_2_@4-ABPT/Cu–Ni (Fig. 4c) due to the coating of nanoparticles by 4-ABPT.

**Figure 5:**
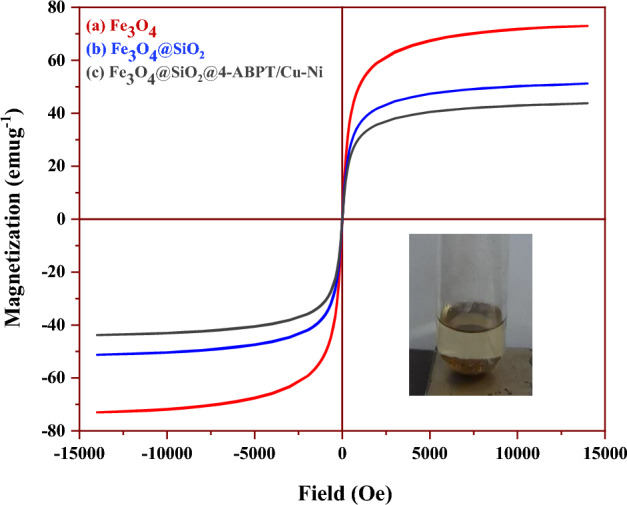
VSM analysis of (**a**) Fe_3_O_4_, (**b**) Fe_3_O_4_@SiO_2_ and (**c**) Fe_3_O_4_@SiO_2_@4-ABPT/Cu–Ni.

The magnetic properties of Fe_3_O_4_, Fe_3_O_4_@SiO_2_, and Fe_3_O_4_@SiO_2_@4-ABPT/Cu–Ni were investigated at room temperature using the vibrating sample magnetometer (VSM) technique (Fig. 5). The S-like magnetization curves with zero magnetic hysteresis loops suggest the superparamagnetic behavior of the samples. As illustrated in Fig. 5, the saturation magnetization (Ms) values of Fe_3_O_4_, Fe_3_O_4_@SiO_2_, and Fe_3_O_4_@SiO_2_@4-ABPT/Cu–Ni NPs are 73, 51.2, and 43.8 emu g^−1^, respectively (Fig. 5a–c). High magnetization of the nanoparticles implies that they could be easily separated from the reaction media using a magnet. Furthermore, the decrease in the saturation magnetization of Fe_3_O_4_@SiO_2_ and Fe_3_O_4_@SiO_2_@4-ABPT/Cu–Ni can be due to the coated silica-shell or Cu/Ni-triazole complex on the surface of Fe_3_O_4_ (Fig. 5b,c).

**Figure 6:**
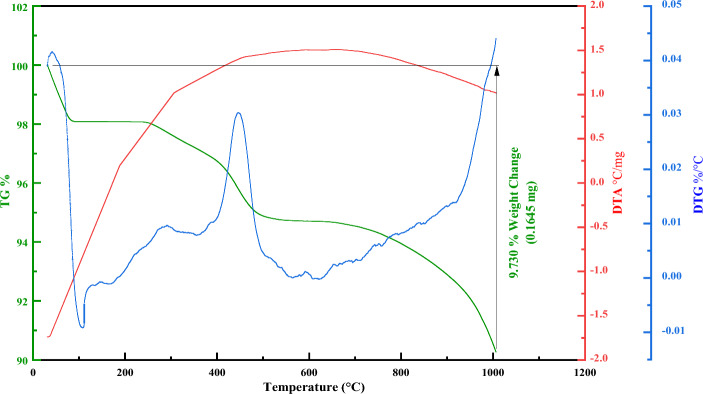
TG-DTG-DTA curves of Fe_3_O_4_@SiO_2_@4-ABPT/Cu–Ni.

Thermogravimetric analysis (TGA), differential thermogravimetric (DTG), and differential thermal analysis (DTA) techniques were utilized to investigate the thermal behavior of Fe_3_O_4_@SiO_2_@4-ABPT/Cu–Ni and confirm the presence of functional groups on the surface of Fe_3_O_4_ nanoparticles. The TGA pattern shown in Fig. 6 exhibits three weight-loss steps in the catalyst. A slight weight loss of about 2% at temperatures below 100 °C can be assigned to the loss of the physically adsorbed water and residual organic solvents. Good thermal stability was seen at 100–250 °C, probably due to the strong chemical interactions between the SiO_2_ coating layer, organic groups, and the Fe_3_O_4_ NPs. Other weight loss steps with an overall loss of about 3% at around 250–500 °C can be attributed to the thermal decomposition of supported organic moieties on the surface of Fe_3_O_4_@SiO_2_ NPs. The complete decomposition of Fe_3_O_4_@SiO_2_@4-ABPT/Cu–Ni, along with the possible phase transition of Fe_3_O_4_ to γ and α-Fe_2_O_3_, occurred at temperatures beyond 650 °C^58–60^. In general, the total weight loss of Fe_3_O_4_@SiO_2_@4-ABPT/Cu–Ni was about 9.73%, suggesting little destruction and excellent heat resistance.

**Figure 7:**
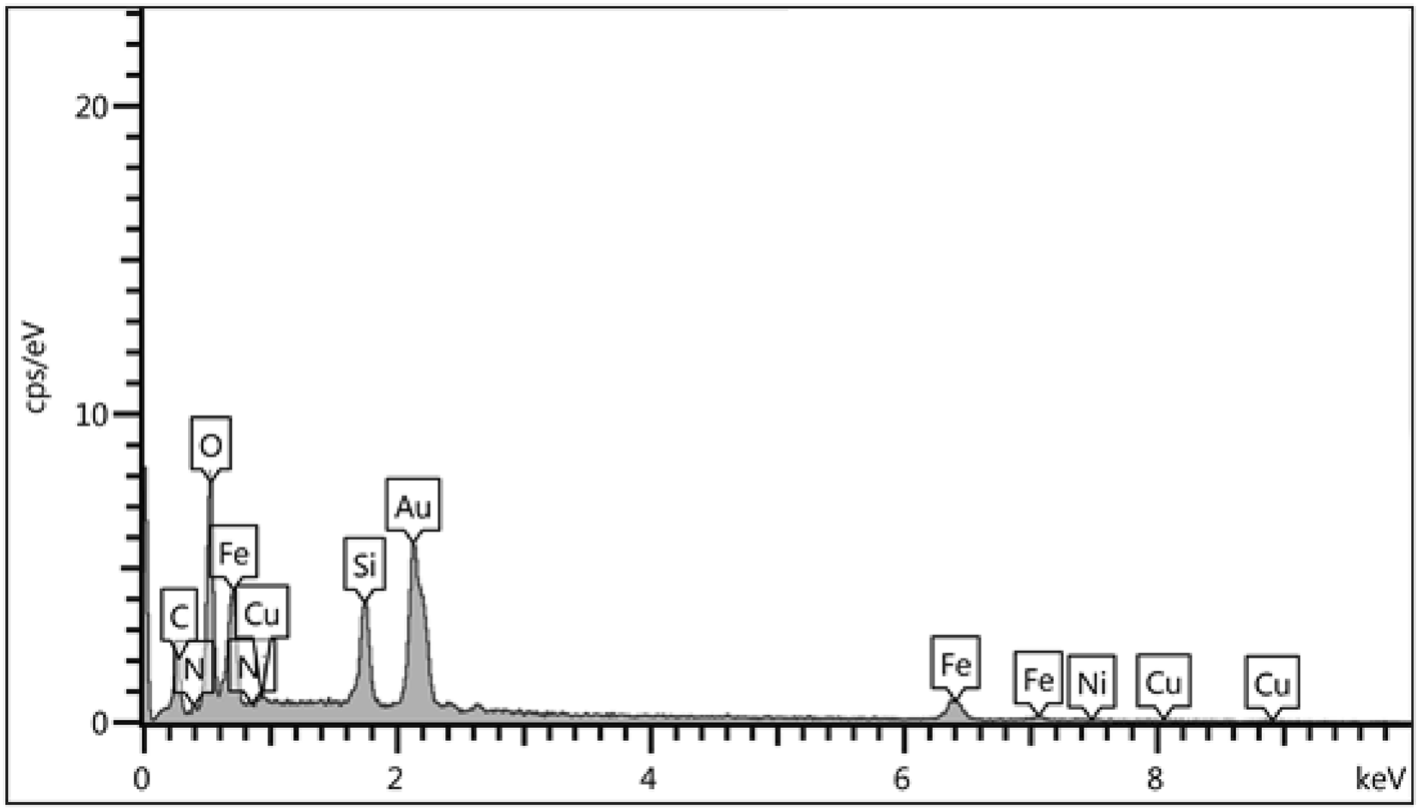
EDX analysis of Fe_3_O_4_@SiO_2_@4-ABPT/Cu–Ni.

The chemical composition of Fe_3_O_4_@SiO_2_@4-ABPT/Cu–Ni was determined by EDX analysis (Fig. 7). The results proved the existence of the expected elements in the structure of Fe_3_O_4_@SiO_2_@4-ABPT/Cu–Ni, namely Si, C, N, O, Fe, Ni, and Cu.

**Figure 8:**
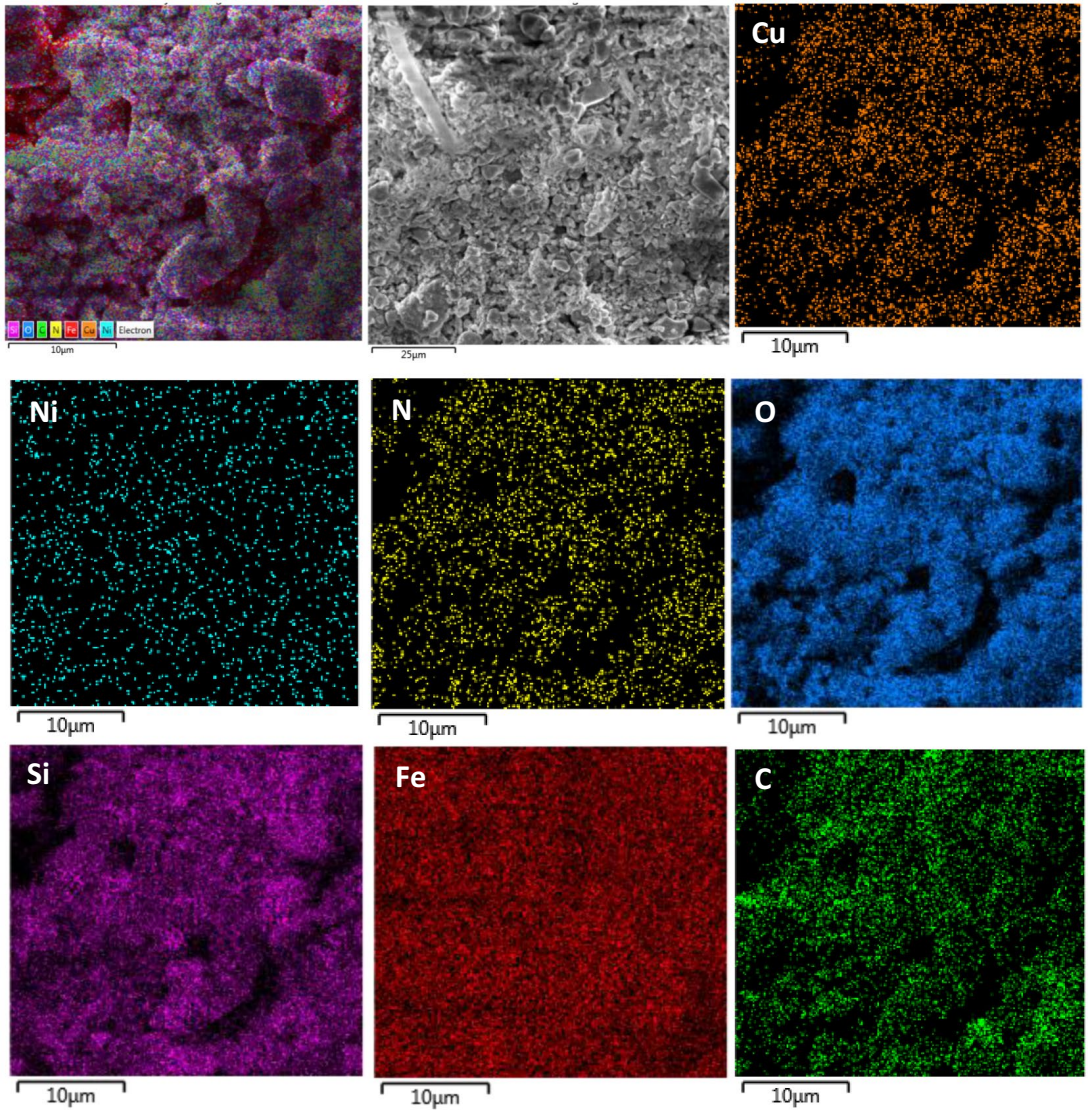
EDS elemental mappings of Fe_3_O_4_@SiO_2_@4-ABPT/Cu–Ni.

To better show the elements on the surface of Fe_3_O_4_@SiO_2_@4-ABPT/Cu–Ni, energy dispersive X-ray spectroscopy (EDS) mapping analysis was taken (Fig. 8). EDS elemental mapping of Fe_3_O_4_@SiO_2_@4-ABPT/Cu–Ni confirmed the presence of all expected elements (Fe, Si, N, C, Ni, Cu, and O), and shows that all of them are evenly distributed over Fe_3_O_4_@SiO_2_@4-ABPT/Cu–Ni.

**Figure 9:**
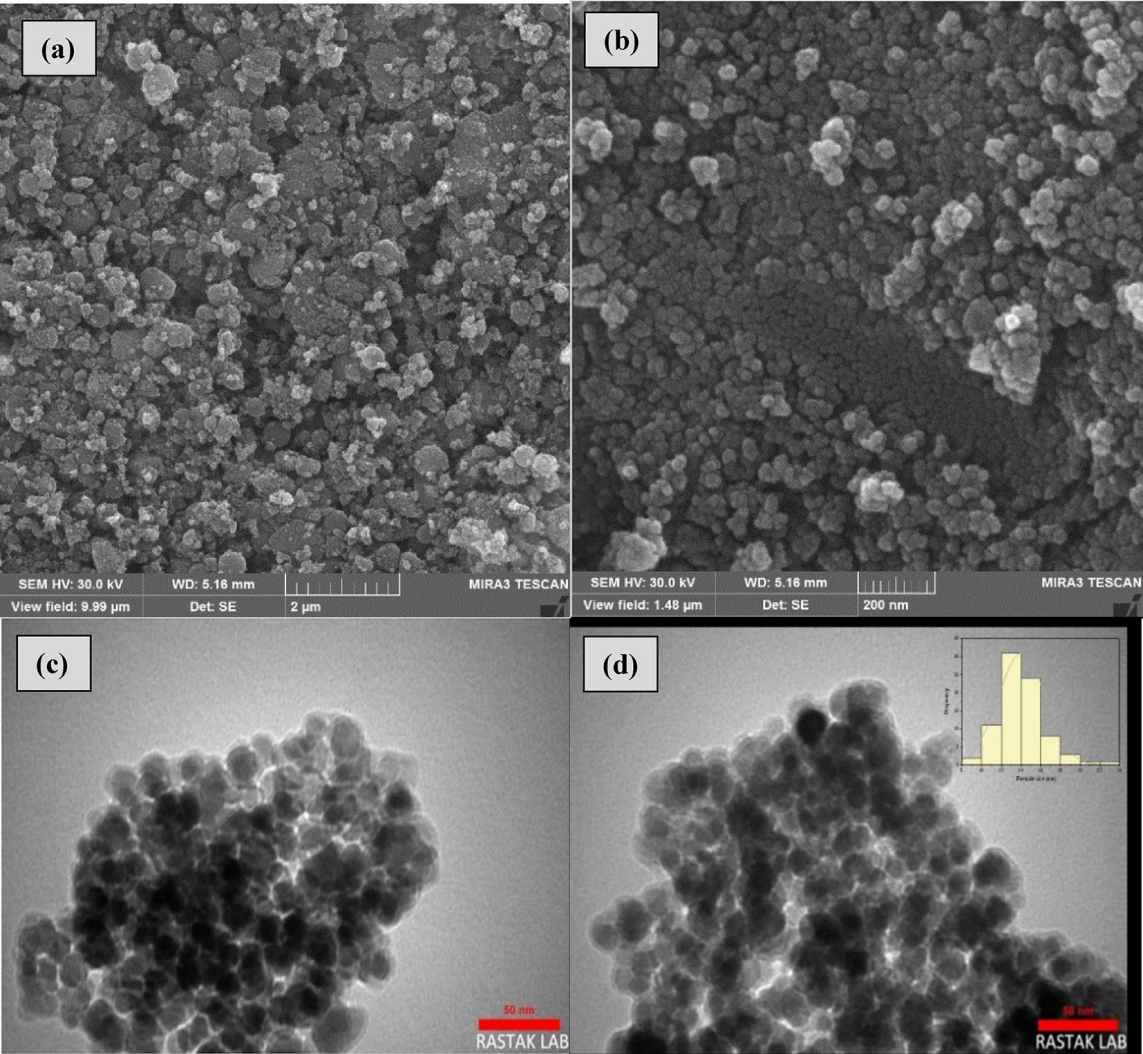
FE-SEM images of (**a** and **b**) Fe_3_O_4_@SiO_2_@4-ABPT/Cu–Ni and TEM images of (**c** and **d**) Fe_3_O_4_@SiO_2_@4-ABPT/Cu–Ni.

FE-SEM and TEM images of Fe_3_O_4_@SiO_2_@4-ABPT/Cu–Ni are shown in Fig. 9(a-d). In Fig. 9, a and b are FE-SEM images of Fe_3_O_4_@SiO_2_@4-ABPT/Cu–Ni, and c and d are TEM images of Fe_3_O_4_@SiO_2_@4-ABPT/Cu–Ni. All four images revealed the spherical morphology and uniform size of Fe_3_O_4_@SiO_2_@4-ABPT/Cu–Ni NPs. Another interesting feature of the TEM images is that Fe_3_O_4_@SiO_2_@4-ABPT/Cu–Ni NPs were highly dispersed. TEM images confirm the core–shell structure with an average size of 14 nm (Fig. 9c,d).

The Cu and Ni contents of the bimetallic catalyst (Fe_3_O_4_@SiO_2_@4-ABPT/Cu–Ni) were calculated and quantified by an inductively coupled plasma analyzer (ICP). The exact Cu and Ni contents were estimated to be 0.08 and 0.06 mmol g^−1^, respectively, suggesting the successful loading of Ni and Cu on the modified magnetic nanoparticles.

**Figure 10:**
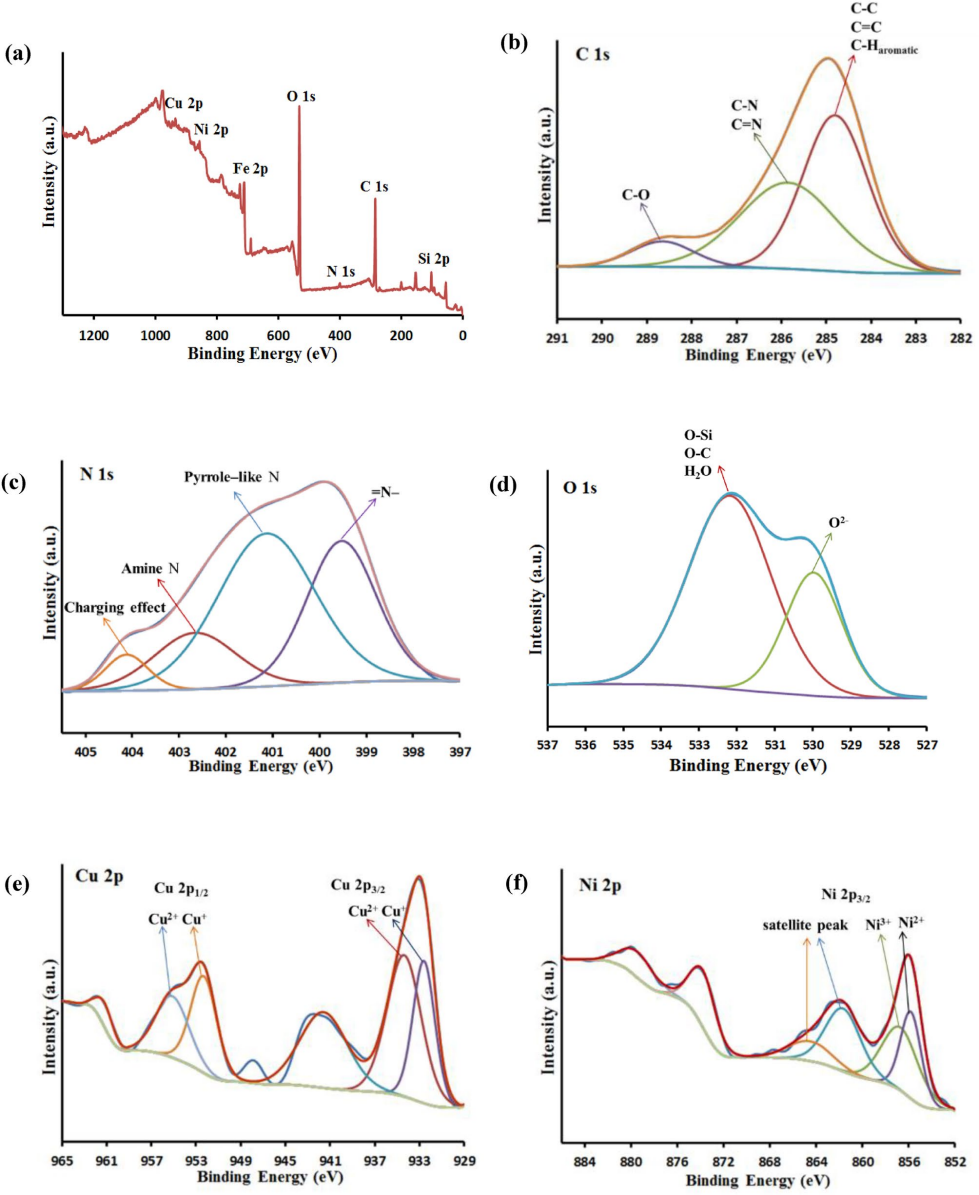
(**a**) XPS survey spectrum of Fe_3_O_4_@SiO_2_@4-ABPT/Cu–Ni; high-resolution XPS spectra of (**b**) C 1s, (**c**) N 1s, (**d**) O 1s, (**e**) Cu 2p, and (**f**) Ni 2p of Fe_3_O_4_@SiO_2_@4-ABPT/Cu–Ni.

To describe the chemical composition of Fe_3_O_4_@SiO_2_@4-ABPT/Cu–Ni surface, a detailed XPS analysis was carried out (Fig. 10). The survey spectrum (Fig. 10a) proved the presence of carbon, nitrogen, silicon, iron, oxygen, nickel, and copper elements on the surface of Fe_3_O_4_@SiO_2_@4-ABPT/Cu–Ni. The C 1s spectrum showed three main peaks (Fig. 10b). The first peak, located at approx. 284.8 eV, can be attributed to the C–C, C=C, and C–H aromatic bonds; the second peak at about 286.0 eV may be assigned to the C–N and C=N bonds; finally, the third peak at higher binding energy (located at approx. 288.7 eV) may be ascribed to the carbon atom of the C–O bond in the methoxy group (–OCH_3_)^61,62^. The N 1s XPS spectrum was deconvoluted into four peaks centered at around 399.6, 401.1, 402.6, and 404.1 eV (Fig. 10c). The first peak can be attributed to the N atom in = N– structure^63,64^. The second one can be related to the pyrrole-like N^65,66^. The third peak at 402.6 may be attributed to triazole ring bonded amine N (–HN–C–)^67^. The last peak with weak intensity (404.1 eV) may be ascribed to the charging effect of triazole rings^68^. The deconvolution of the O 1s region showed two peaks (Fig. 10d). The first peak (located at approx. 530.1 eV) can be related to the lattice oxygen (O^2−^) in the Fe_3_O_4_; the second peak at about 532.3 eV may be assigned to the oxygen of methoxy group (–OCH_3_), O-Si, and adsorbed water^61,62,69^. Figure 10e shows the Cu 2*p* XPS spectra of the Fe_3_O_4_@SiO_2_@4-ABPT/Cu–Ni nanocatalyst. The Cu 2p_3/2_ and Cu 2p_1/2_ binding energies are represented by two couples of peaks at 932.5 and 934.7 1 eV and 952.7 and 954.8 eV, reflecting the coexistence of Cu(I) and Cu(II) sites in Fe_3_O_4_@SiO_2_@4-ABPT/Cu–Ni. In most cases, the spin–orbit splitting of the 2p_3/2_ and 2p_1/2_ peaks of Ni-containing compounds is large enough, so only the more intense 2p_3/2_ signal needs to be considered^27^. The spectrum of Ni 2p_3/2_ (Fig. 10f) exhibits binding energies at 855.9 and 856.7 eV corresponding to the nickel centers with + 2 and + 3 oxidation states, respectively. The accompanying satellite peaks of Ni 2p_3/2_ were observed at around 862 and 864.8 eV^70,71^. The relative amounts of Cu(I) and Ni(III) can be estimated by the integration of the peaks. As can be seen in Fig. 10e,f, the peak intensities of Cu(I) and Ni(III) are nearly equivalent to those of Cu(II) and Ni(II), respectively. The presence of conjugated ligand as a bridge in the bimetallic system helps to promote electron transfer between the Cu and Ni centers, strengthening the synergistic effect and promoting the conversion of more Cu(II) and Ni(II) into active Cu(I) and Ni(III), respectively^46^.

## Optimization of reaction parameters

After the successful synthesis and characterization of Fe_3_O_4_@SiO_2_@4-ABPT/Cu–Ni catalyst, its catalytic activity was probed in the Sonogashira C–C coupling reaction. The reaction parameters were optimized by the cross-coupling between aryl iodide and phenylacetylene as a simple model reaction. Several reaction factors, such as base type, temperature, solvent, and catalyst content were screened (Table 1). Based on Table 1, a range of polar and non-polar solvents was first investigated (Table 1, entries 1–12). However, an excellent yield of the desired product (85%) was obtained when the reaction was performed without solvent (Table 1, entry 13). Next, different amounts of Fe_3_O_4_@SiO_2_@4-ABPT/Cu–Ni were examined in the model reaction (Table 1, entries 14–16). For this transformation, 0.01g of the catalyst containing 0.06 mol% Ni and 0.08 mol% Cu led to the best results (Table 1, entry 14). A further increase in the catalyst concentration substantially did not improve the yield (Table 1, entry 16). The effects of reaction temperature were also studied in the Sonogashira cross-coupling (Table 1, entries 17–20), where the best results were achieved at 120 °C (Table 1, entry 20). A lower yield was obtained when the reaction temperature was reduced (Table 1, entries 17–18). Finally, due to the significant role of bases in coupling reactions, various strong and weak bases were tested under identical reaction conditions (Table 1, entries 21–26). The best yield was obtained with NaO^*t*^Bu (Table 1, entry 26). Accordingly, NaO^*t*^Bu as the base, solvent-free conditions, 0.01 g of Fe_3_O_4_@SiO_2_@4-ABPT/Cu–Ni (0.06 mol% Ni, 0.08 mol% Cu) as the amount of catalyst, and 120 °C as the optimum temperature was found to be the optimal conditions for Fe_3_O_4_@SiO_2_@4-ABPT/Cu–Ni-catalyzed Sonogashira C–C coupling reactions (Table 1).

**Table 1:** Optimization of the reaction parameters for Sonogashira cross-coupling of phenylacetylene and iodobenzene catalyzed by Fe_3_O_4_@SiO_2_@4-ABPT/Cu–Ni NPs.

Entry^a^	Solvent	Catalyst (g)	Temp. (°C)	Base	Time (h)	Yield^b^ (%)
1	H_2_O	0.008	Reflux	NaOH	6	22
2	EtOH	0.008	Reflux	NaOH	4	80
3	CHCl_3_	0.008	Reflux	NaOH	3.5	81
4	CH_2_Cl_2_	0.008	Reflux	NaOH	5.5	66
5	CH_3_CN	0.008	Reflux	NaOH	4.5	70
6	THF	0.008	Reflux	NaOH	2	75
7	Toluene	0.008	Reflux	NaOH	3.5	31
8	DMSO	0.008	100	NaOH	2.5	76
9	MeOH	0.008	Reflux	NaOH	3	80
10	Dioxane	0.008	Reflux	NaOH	2.5	80
11	DMF	0.008	100	NaOH	3	74
12	EtOAc	0.008	Reflux	NaOH	5.5	60
13	none	0.008	100	NaOH	2	85
14	none	0.01	100	NaOH	1.5	86
15	none	0.005	100	NaOH	2	75
16	none	0.015	100	NaOH	1.5	87
17	none	0.01	70	NaOH	3.5	80
18	none	0.01	90	NaOH	2	82
19	none	0.01	140	NaOH	1	88
20	none	0.01	120	NaOH	1	88
21	none	0.01	120	KOH	1	83
22	none	0.01	120	NaOAc	3	75
23	none	0.01	120	K_2_CO_3_	4.5	75
24	none	0.01	120	K_3_PO_4_	3	80
25	none	0.01	120	Et_3_N	5	82
26	none	0.01	120	NaO^*t*^Bu	1	95

^a^Reaction conditions: phenylacetylene (1.5 mmol), iodobenzene (1.0 mmol), base (1 mmol), solvent (2 mL). ^b^Isolated yield.

Under the optimized conditions, the scope of the Sonogashira cross-couplings was surveyed using various aryl halides and phenylacetylene in the presence of Fe_3_O_4_@SiO_2_@4-ABPT/Cu–Ni nanocatalyst (Table 2). As shown in Table 2, three aryl iodides, bromides, and chlorides effectively reacted with phenylacetylene to produce the corresponding products at high to excellent yields (Table 2, 70–95%). It should be noted that the Sonogashira reaction was highly selective as no Glaser-type homo-coupling or other side-coupling product was observed. Generally, aryl halides with electron-withdrawing functional groups such as –NO_2_ and –CHO provided higher (produced better) efficiencies than those encompassing electron-donating substituents such as –OMe and –Me (Table 2, entries 2–4 versus 6–8). It means that a decline in the electron density of the aromatic ring accelerated the halide elimination from the substrate in the oxidative-addition step of the Sonogashira cross-coupling reaction^72^. Moreover, the reactions of aryl iodides were slightly faster than their chloro and bromo analogs due to the lower C–I bond strength compared to C–Br and C–Cl bonds (C–Cl > C–Br > C–I)^73^. This order of reactivity, where aryl iodides are more reactive than aryl bromides and then aryl chlorides, is also in agreement with an oxidative addition/reductive elimination mechanism in which the rate-determining step is the breaking of the bond to the leaving group^4^.

**Table 2:** Sonogashira cross-coupling reaction of phenylacetylene with various aryl halides catalyzed by Fe_3_O_4_@SiO_2_@4-ABPT/Cu–Ni NPs.


Entry^a^	R	X	Time (h)	Yield^b^ (%)
1	H	I	1	95
2	4-Me	I	1.5	76
3	2-Me	I	2.5	73
4	4-OMe	I	3	75
5	4-CO_2_H	I	1.5	87
6	4-NO_2_	I	1	82
7	2-NO_2_	I	1.5	80
8	4-COH	I	2	90
9	H	Br	1.5	90
10	4-NH_2_	Br	4.5	74
11	4-CO_2_H	Br	3	76
12	4-CN	Br	3.5	85
13	H	Cl	2.5	95
14	2-NH_2_	Cl	4.5	75
15	3-NH_2_	Cl	4	70
16	4-NH_2_	Cl	4.5	78

^a^Reaction conditions: aryl halide (1.0 mmol), phenylacetylene (1.5 mmol), NaO^*t*^Bu (1.0 mmol), solvent-free, catalyst (0.01 g, 0.06 mol% Ni, 0.08 mol% Cu), and 120 °C. ^b^Isolated yield.

Due to the excellent results obtained from the Sonogashira cross-coupling reaction and the high performance of Fe_3_O_4_@SiO_2_@4-ABPT/Cu–Ni nanocatalyst, under the optimum conditions obtained for the Sonogashira reaction, the carbon–nitrogen cross-couplings were also investigated. For this purpose, reactions were carried out at 120 °C in a solvent-free environment using Fe_3_O_4_@SiO_2_@4-ABPT/Cu–Ni (0.01 g, 0.08 mol% Cu, 0.06 mol% Ni) and various aryl halides as well as phenylboronic acids with *N*-heterocyclic compounds. When compared to aryl halide derivatives, it was found that phenylboronic acid significantly influences the desired output product (Table 3, entries 1–6). The electron-withdrawing haloarenes react more quickly with imidazole than electron-donating haloarenes (Table 3, entries 7 vs. 8).

**Table 3:**
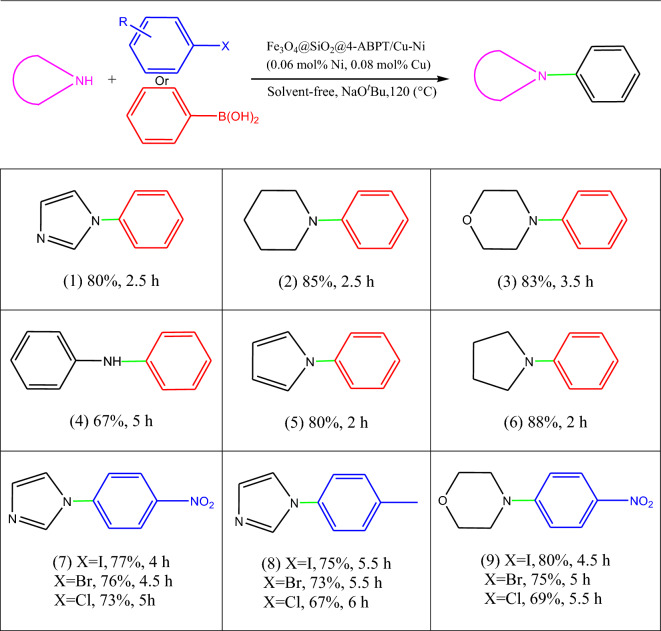
C–N cross-coupling reaction of phenylboronic acid or aryl halides with *N*-heterocyclic compounds catalyzed by Fe_3_O_4_@SiO_2_@4-ABPT/Cu–Ni NPs^a,b^.

^a^Reaction conditions: aryl halide or phenylboronic acid (1 mmol), *N*-heterocyclic compound (1.3 mmol), NaO^*t*^Bu (1.0 mmol), solvent-free, catalyst (0.01 g, 0.06 mol% Ni, 0.08 mol% Cu), and 120 °C. ^b^Isolated yield.

## Control experiments

The high catalytic performance of Fe_3_O_4_@SiO_2_@4-ABPT/Cu–Ni was elucidated by performing several control experiments, in which the catalytic activity of different species was studied for the Sonogashira cross-coupling model reaction under optimum conditions (Table 4). For a better comparison, the reaction time was considered constant. The corresponding results are summarized in Table 4. The results showed that the catalytic activity of Fe_3_O_4_@SiO_2_@4-ABPT/Cu–Ni was unique compared to the other catalyst components alone: (1) Reaction in the absence of the catalyst showed no detectable efficiency (Table 4, entry 1), (2) Fe_3_O_4_ and Fe_3_O_4_@SiO_2_ magnetic nanoparticles did not display any catalytic effect (Table 4, entries 2,3), (3) As shown in Table 4, entry 4, no catalytic activity was observed for Fe_3_O_4_@SiO_2_@4-ABPT NPs in the coupling model reaction, (4) By coordinating copper (Fe_3_O_4_@SiO_2_@4-ABPT/Cu) or nickel (Fe_3_O_4_@SiO_2_@4-ABPT/Ni) metals, the yield increased to 65% and 35%, respectively (Table 4, entries 5,6). These results manifested the catalytic efficiency of the copper and nickel metal centers for the Sonogashira cross-coupling reaction, (5) Without the 4-ABPT ligand, copper and nickel salts were used, but no remarkable product was produced (15%, Table 4, entry 7), indicating that the 4-ABPT ligand may be crucial for Fe_3_O_4_@SiO_2_@4-ABPT catalytic system. (6) When a physical mixture of Fe_3_O_4_@SiO_2_@4-ABPT/Cu and Fe_3_O_4_@SiO_2_@4-ABPT/Ni was used, the yield was 75% (Table 4, entry 8). Significantly, the simultaneous presence of copper and nickel connected with 4-ABPT bridging ligand in the bimetallic nanocatalyst (Fe_3_O_4_@SiO_2_@4-ABPT/Cu–Ni) provided a higher chemical yield (95%) compared to the monometallic counterparts, physical mixture of monometallic counterparts, and non-supported metal salts. The improved catalytic activity of this bimetallic system probably originated from a synergistic cooperative effect between conjugated 4-ABPT ligand, Ni, and Cu metal centers.

**Table 4:** Designed control experiments for our Sonogashira cross-coupling reaction.

Entry^a^	Catalyst	Yield^g^ (%)
1	No catalyst	No reaction
2	Fe_3_O_4_	No reaction
3	Fe_3_O_4_@SiO_2_	No reaction
4	Fe_3_O_4_@SiO_2_@4-ABPT	No reaction
5^b^	Fe_3_O_4_@SiO_2_@4-ABPT/Cu	65
6^c^	Fe_3_O_4_@SiO_2_@4-ABPT/Ni	35
7^d^	Cu(OAc)_2_. H_2_O + Ni(OAc)_2_.4H_2_O	15
8^e^	Fe_3_O_4_@SiO_2_@4-ABPT/Cu + Fe_3_O_4_@SiO_2_@4-ABPT/Ni	75
9^f^	Fe_3_O_4_@SiO_2_@4-ABPT/Cu–Ni	95

^a^Reaction conditions: phenylacetylene (1.5 mmol), iodobenzene (1.0 mmol), NaO^*t*^Bu (1.0 mmol), solvent-free, Cat. (0.01 g), 120 °C, 1 h. ^b^(0.01 g, 0.08 mol% Cu). ^c^(0.01 g, 0.06 mol% Ni). ^d,e^(0.06 mol% Ni, 0.08 mol% Cu). ^f^(0.01 g, 0.06 mol% Ni, 0.08 mol% Cu). ^g^Isolated yield.

To better illustrate the merits of Fe_3_O_4_@SiO_2_@4-ABPT/Cu–Ni for the C–C and C–N cross-coupling reactions, the present catalyst system was compared with several previously reported bimetallic catalysts. The results are shown in Table 5. The Fe_3_O_4_@SiO_2_@4-ABPT/Cu–Ni catalyst showed several superiorities over other reports such as short reaction time, high efficiency, low catalyst loading, low metal toxicity, absence of toxic organic solvents, and simple catalyst recycling.

**Table 5:** Comparison of catalyst efficiency of Fe_3_O_4_@SiO_2_@4-ABPT/Cu–Ni with other methods described in the literature.

Entry	Reaction	Catalyst	Condition	Time (h)	Yield (%)
1^This work^	Sonogashira^a^	Fe_3_O_4_@SiO_2_@4-ABPT/Cu–Ni (0.06 mol% Ni, 0.08 mol% Cu)	Solvent-free/120 °C/NaO^*t*^Bu	1	95
2^74^		Pd–Cu–PA^b^ (0.1 mol% Pd)	NMP/100 °C/Bu_3_N	5	88
3^75^		PdCu@GQD@Fe_3_O_4_^c^ (Pd 0.3 mol%, Cu 0.35 mol%)	DABCO/Toluene/50 °C	24	99
4^76^		Pd/Cu@MCC-PAMAM-PEI^d^ (0.65 mol% Pd, 2.55 mol% Cu)	DMSO/80 °C/K_2_CO_3_/N_2_ atmosphere	4	96
5^77^		Cu/Pd@Mod-PANI-3OH^e^ (0.094 mol% Pd)	H_2_O/80 °C /Et_3_N	8	90
6^78^		Fe_3_O_4_@PEG/Cu–Co^f^ (0.13 mol% Co, 0.37 mol% Cu)	H_2_O/80 °C/base-free	3.5	95
7^This work^	C–N coupling^g^	Fe_3_O_4_@SiO_2_@4-ABPT/Cu–Ni (0.06 mol% Ni, 0.08 mol% Cu)	Solvent-free/120 °C/NaO^*t*^Bu	4.5	80
8^79^		Pd (I) dimer^h^ (2 mol% Pd)	Toluene/40 °C/NaO^*t*^Bu/AgOTf	4	71
9^78^		Fe_3_O_4_@PEG/Cu–Co (0.17 mol% Co, 0.47 mol% Cu)	H_2_O/80 °C/base-free	7	75

^a^Sonogashira reaction of iodobenzene and phenylacetylene. ^b^PA = Polyamide ligand. ^c^GQD = Graphene quantum dot. ^d^MCC-PAMAM-PEI = Polyethyleneimine end-capped microcrystalline cellulose-polyamidoamine dendrimer. ^e^Mod-PANI-3OH = Modified polyaniline. ^f^PEG = Polyethylene glycol. ^g^C–N coupling reaction of 1-iodo-4-nitrobenzene and morpholine. ^h^Phosphinoimidazole-derived Pd(I) complex.

## Mechanistic study

According to the results of control reactions, the simultaneous presence of nickel and copper centers connected with 4-ABPT has a synergistic effect, increasing catalyst efficiency significantly. The synergetic interactions between active metal sites may be enhanced by an organic linker that bridges different metal centers and plays a sensitive charge transfer role^80^.

**Figure 11:**
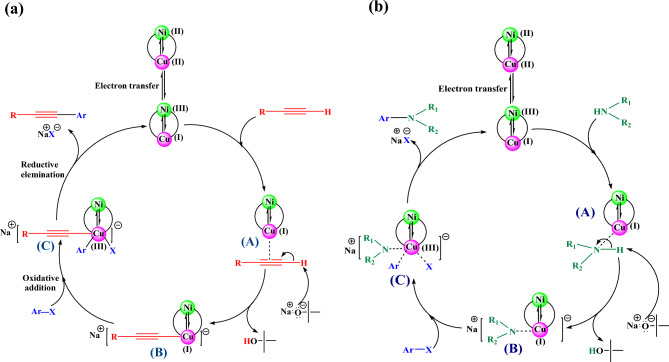
A plausible reaction mechanism for Fe_3_O_4_@SiO_2_@4-ABPT/Cu–Ni-catalyzed (**a**) Sonogashira and (**b**) C–N cross-coupling reactions.

In General, our observations and reported mechanisms suggest a plausible mechanism for the Fe_3_O_4_@SiO_2_@4-ABPT/Cu–Ni-catalyzed Sonogashira cross-coupling reaction based on oxidative addition and reductive elimination steps^78,81,82^. It is impossible to certainly recognize which metal carries out the reaction via oxidative addition and reductive elimination ^78^. However, a proposed mechanism on the basis of our results and previous reports is outlined in Fig. 11, in which the copper metal is suggested to perform the reaction. In the first step, electron transfer from Ni^2+^ through the 4-ABPT ligand led to the reduction of Cu^2+^ to Cu^+^. Then, a π-complex is created between the acetylene groups and the metal centers of the catalyst. Cu^+^ activates phenylacetylene by generating transient copper acetylide intermediate B in the presence of NaO^*t*^Bu. Oxidative addition of aryl halides to Cu^+1^ produces Cu^+3^. Finally, a reductive elimination reaction forms the desired coupling product from the resulting intermediate C, and the catalyst returns to the cycle (Fig. 11a).

As shown in Fig. 11b , a similar mechanism can be proposed for the Fe_3_O_4_@SiO_2_@4-ABPT/Cu–Ni-catalyzed C–N cross-coupling reaction. Initially, the nucleophilic attack of the amine group on the formed active Cu(I) species gives intermediate A. The proton is then taken away from the amine by NaO^*t*^Bu, leading to the formation of intermediate B. After this, the oxidative addition of aryl halide close to intermediate B causes intermediate C, and the active centers of Cu(I) are oxidized to Cu(III). Ultimately, a reductive elimination stage resulted in the C–N coupling product and the regeneration of the bimetallic catalyst.

To explore the reaction mechanism more precisely and better investigate the synergistic effect between Ni and Cu species, some analysis was performed.

**Figure 12:**
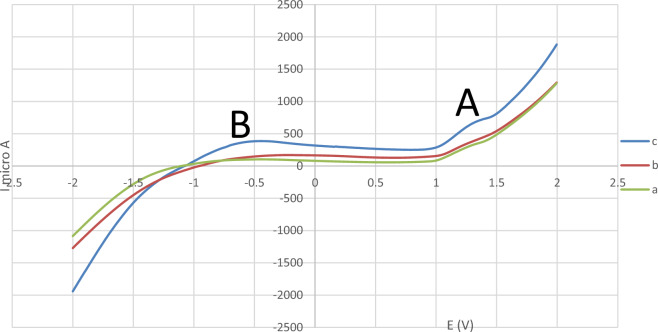
LSV voltammogram of (**a**) Fe_3_O_4_@SiO_2_@4-ABPT/Cu, (**b**) Fe_3_O_4_@SiO_2_@4-ABPT/Ni, and (**c**) Fe_3_O_4_@SiO_2_@4-ABPT/Cu–Ni in 0.1 mol L^-1^ Britton–Robinson (BR) buffer solution (pH 7.0) with a scan rate of 100 mV s^-1^ at room temperature.

The electrochemical behavior of Fe_3_O_4_@SiO_2_@4-ABPT/Cu, Fe_3_O_4_@SiO_2_@4-ABPT/Ni, and Fe_3_O_4_@SiO_2_@4-ABPT/Cu–Ni was studied by linear sweep voltammetry (LSV) technique in the potential range of − 2.0 to + 2.0 V (Fig. 12). The LSV voltammogram of Fe_3_O_4_@SiO_2_@4-ABPT/Cu shows a weak peak at the A area corresponding to Cu(I) ⇌ Cu(II), indicating low electron transfer process (Fig. 12a). The LSV voltammogram of Fe_3_O_4_@SiO_2_@4-ABPT/Ni demonstrates a weak peak at the B area, which is related to Ni(II) ⇌ Ni(III), indicating low electron transfer process (Fig. 12b). In the LSV voltammogram of Fe_3_O_4_@SiO_2_@4-ABPT/Cu–Ni, both peaks at A and B areas, which correspond to Cu(I) ⇌ Cu(II) and Ni(II) ⇌ Ni(III) respectively, exhibit strong current. The peaks intensity of Fe_3_O_4_@SiO_2_@4-ABPT/Cu–Ni is significantly increased compared to the peaks intensity of its monometallic counterparts (Fe_3_O_4_@SiO_2_@4-ABPT/Cu and Fe_3_O_4_@SiO_2_@4-ABPT/Ni), suggesting proper electron transfer due to synergistic effect between the copper and nickel metals in Fe_3_O_4_@SiO_2_@4-ABPT/Cu–Ni.

**Figure 13:**
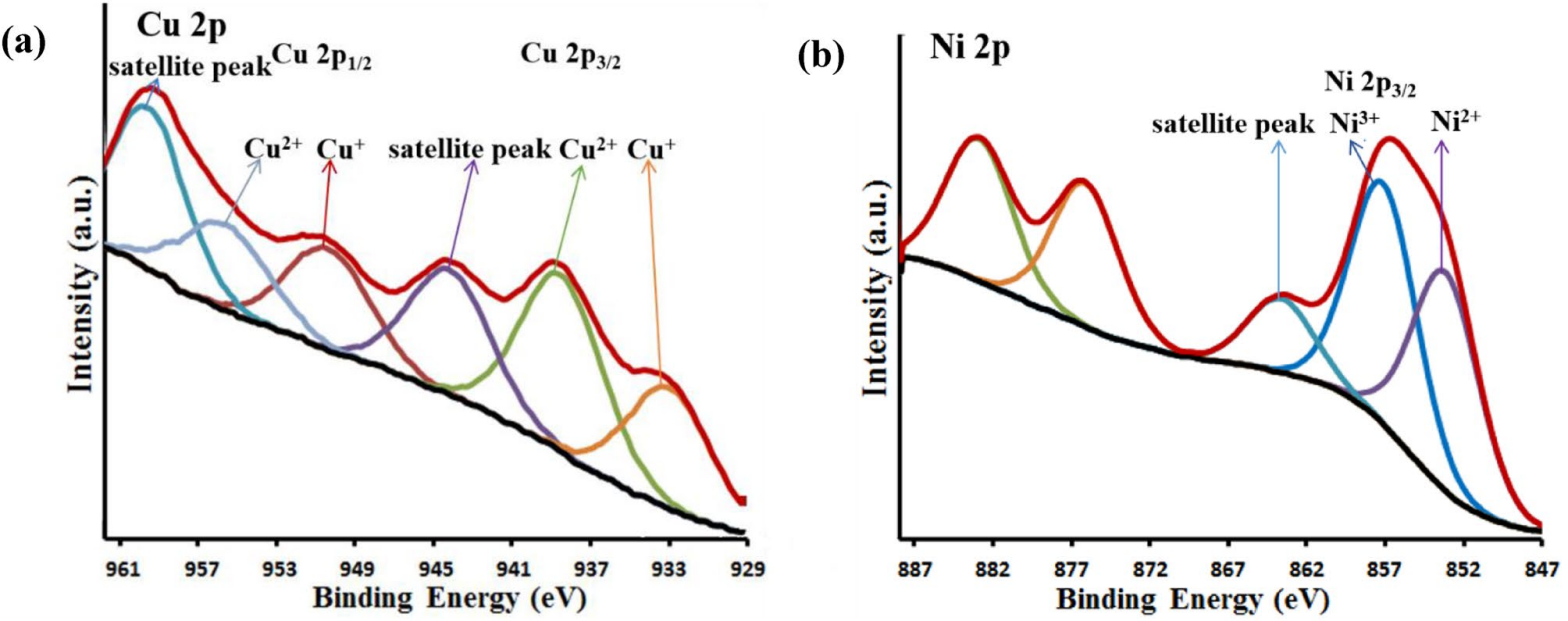
The high-resolution XPS spectra of (**a**) Cu 2p and (**b**) Ni 2p of recovered Fe_3_O_4_@SiO_2_@4-ABPT/Cu–Ni.

Considering the case of the Sonogashira reaction, XPS analysis of the recovered catalyst was performed (Fig. 13). Figure 13a shows the high-resolution spectra of Cu 2p for recovered Fe_3_O_4_@SiO_2_@4-ABPT/Cu–Ni nanocatalyst. The peak area ratio of Cu^+^/Cu^2+^ was 0.8, while this ratio was 0.7 for the fresh catalyst. The increase in Cu^+^ production suggests that Cu^+^ might be the active center during the catalytic process. Noteworthy, the peak position of Cu^+^ at 2p_1/2_ was significantly shifted to lower binding energy (951 eV) compared with that of fresh catalyst (952.7 eV, Fig. 10e), indicating that the Ni atoms contribute to increasing the electronic density of Cu centers and facilitates the oxidative addition of haloarenes to Cu(I)^83,84^. Figure 13b shows the high-resolution XPS spectra of Ni 2p for used Fe_3_O_4_@SiO_2_@4-ABPT/Cu–Ni. A slight positive shift can be seen to higher binding energy (857.5 eV) for Ni^3+^ relative to that of the unused catalyst (856.7, Fig. 10f), indicating that electron might transfer from Ni to Cu occurred due to the cooperation between Ni and Cu^85,86^. All these points helped to explain the existence of interactions between copper and nickel species.

Based on the results of the above analyses, the proposed mechanism and synergistic effect between copper and nickel metal centers can be verified.

## Recoverability studies

**Figure 14:**
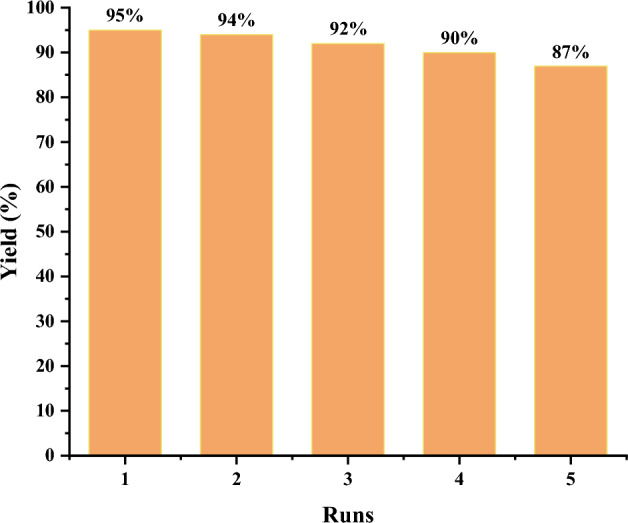
Recyclability of Fe_3_O_4_@SiO_2_@4-ABPT/Cu–Ni NPs in the Sonogashira reaction of iodobenzene (1 mmol) with phenylacetylene (1.5 mmol) in the presence of Fe_3_O_4_@SiO_2_@4-ABPT/Cu–Ni (0.01 g, 0.06 mol% Ni, 0.08 mol% Cu), and NaO^*t*^Bu (1.0 mmol) at 120 °C for 1 h under solvent-free conditions.

Based on sustainable and green chemistry principles, catalyst reusability and stability are critical factors in evaluating the efficiency of a heterogeneous catalyst. In this way, we studied the recovery and recycling of Fe_3_O_4_@SiO_2_@4-ABPT/Cu–Ni nanocatalyst in the model reaction of Sonogashira cross-coupling under optimal reaction conditions (Fig. 14). As shown in Fig. 14, Fe_3_O_4_@SiO_2_@4-ABPT/Cu–Ni demonstrated a relatively consistent product yield after five successive runs with minimal efficiency loss. The efficiency reached 87% (a mere 8% decrease) after five runs, which is negligible.

**Figure 15:**
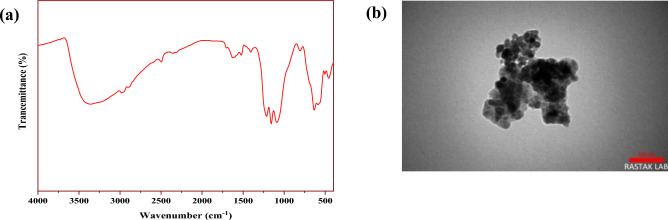
FT-IR spectrum (**a**) and TEM image (**b**) of the reused Fe_3_O_4_@SiO_2_@4-ABPT/Cu–Ni nanocatalyst after the 5th run.

FT-IR, TEM, and ICP analyses were used to characterize the recovered Fe_3_O_4_@SiO_2_@4-ABPT/Cu–Ni nanocatalyst after the fifth run to determine its stability (Fig. 15a,b). The main structure of the nanocatalyst was preserved after successive recoveries and reuses, as shown by the FT-IR analysis of the recovered catalyst, which was almost identical to that of the fresh sample (Fig. 15a). The TEM image of the recovered Fe_3_O_4_@SiO_2_@4-ABPT/Cu–Ni demonstrated no morphological changes after the 5th cycle (Fig. 15b). Based on ICP analysis, the amount of copper and nickel was 0.073 and 0.051 mmol g^−1^, respectively, suggesting negligible copper and nickel leaching (the Cu and Ni contents of the fresh catalyst were 0.08 and 0.06 mmol g^−1^, respectively).

## Conclusion

**Figure 16:**
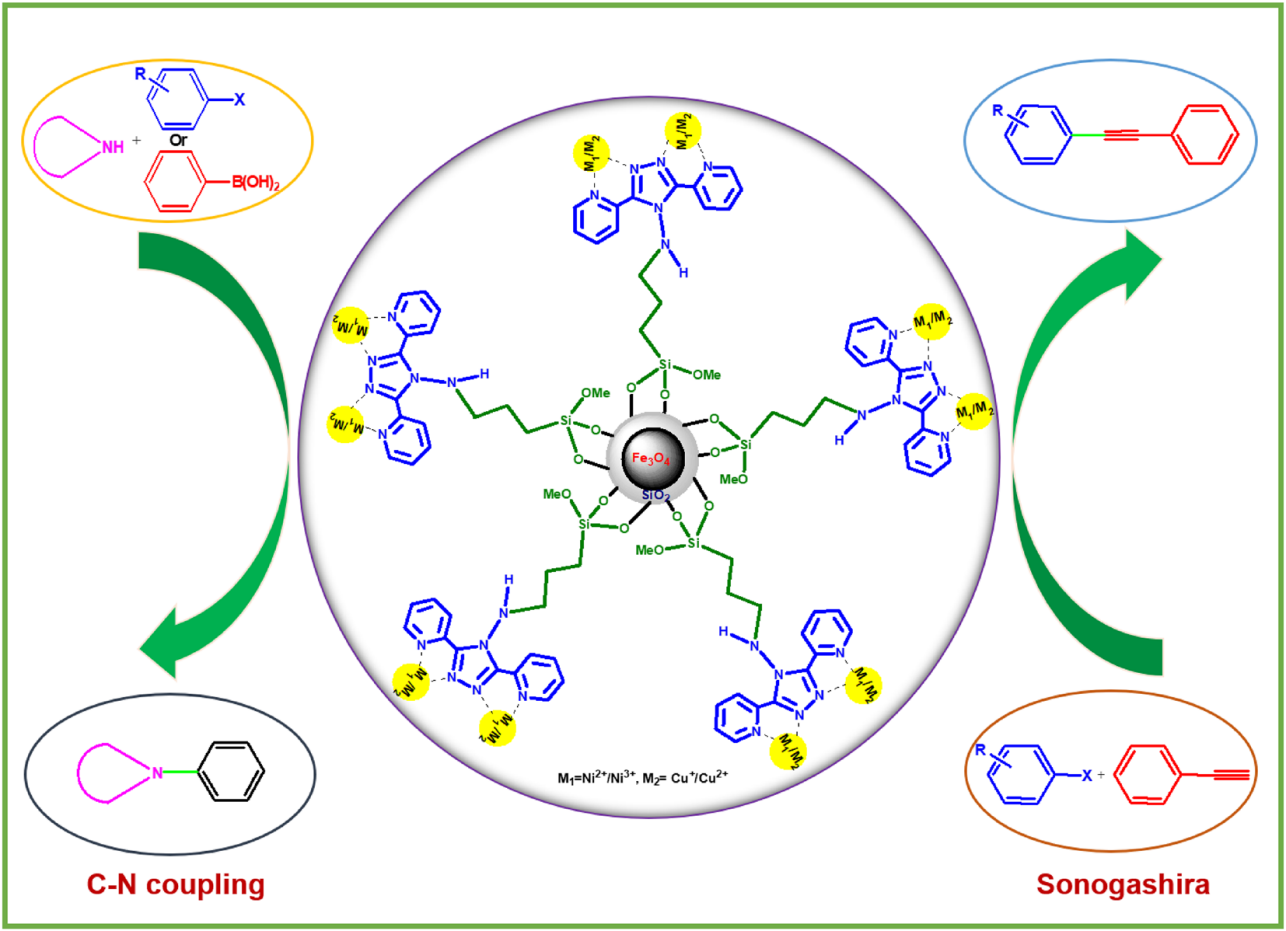
Graphical abstract.

Briefly, this research reports the synthesis of a new magnetically recyclable Cu–Ni bimetallic system containing π-conjugated 4-ABPT bridging ligand (Fe_3_O_4_@SiO_2_@4-ABPT/Cu–Ni). Fe_3_O_4_@SiO_2_@4-ABPT/Cu–Ni was then characterized by FT-IR, XRD, EDX-mapping, LSV, FE-SEM, TEM, TGA, ICP, VSM, and XPS analyses. The developed nanocatalyst served well in the selective C–N and Sonogashira cross-coupling reactions under Pd- and solvent-free conditions. Fe_3_O_4_@SiO_2_@4-ABPT/Cu–Ni nanocatalyst displayed high catalytic performance for various substrates despite its low copper and nickel contents (0.06 mol% Ni, 0.08 mol% Cu). By running the C–N and Sonogashira cross-coupling reactions with monometallic counterparts (Fe_3_O_4_@SiO_2_@4-ABPT/Cu and Fe_3_O_4_@SiO_2_@4-ABPT/Ni), the synergic effect of Fe_3_O_4_@SiO_2_@4-ABPT/Cu–Ni as a conjugated bimetallic system can be revealed. According to the XPS results for the peak intensities of the Cu and Ni species in Fe_3_O_4_@SiO_2_@4-ABPT/Cu–Ni, an electron can be transferred from Ni(II) to Cu(II) through the conjugated 4-ABPT ligand to form the active Cu(I) species. The copper center of Fe_3_O_4_@SiO_2_@4-ABPT/Cu–Ni is suggested to carry out the cross-coupling processes via an oxidative addition/reductive elimination pathway. The catalytic synergy between ligand, Ni, and Cu led to high activity and selectivity of Fe_3_O_4_@SiO_2_@4-ABPT/Cu–Ni as a catalyst in the cross-coupling transformations. Furthermore, the magnetic properties of Fe_3_O_4_@SiO_2_@4-ABPT/Cu–Ni nanoparticles promoted its separation and reuse while streamlining the work-up process. FT-IR and TEM analyses of the recovered Fe_3_O_4_@SiO_2_@4-ABPT/Cu–Ni demonstrated its stability after the fifth cycle. These analyses revealed that its proposed structure and morphology were nearly identical to those of the fresh one. Hence, Fe_3_O_4_@SiO_2_@4-ABPT/Cu–Ni nanomaterial can be a promising catalyst for the industrial manufacturing of arylamine and biphenylacetylene derivatives (Fig. 16).

### Supplementary Information

Supplementary Information.

## Data Availability

All data generated or analyzed during this study are included in this published article (and its Supplementary Information files).
