# Supplementary material for: Synergistic performance of a new bimetallic complex supported on magnetic nanoparticles for Sonogashira and C–N coupling reactions
Source: Sci Rep. 2023 Oct 24;13:18153. doi: 10.1038/s41598-023-44168-6 (PMC10598020; doi:10.1038/s41598-023-44168-6)
Supplement: Supplementary file 1 — Supplementary Information. [file 41598_2023_44168_MOESM1_ESM.pdf]

Supplementary Materials

Synergistic performance of a new bimetallic complex supported on magnetic nanoparticles for Sonogashira and C−N coupling reactions

Fatemeh Nasseri[1,2], Mohammad Ali Nasseri[2*], Mohamad Zaman Kassaee[1], Issa Yavari[1]

[1] Department of Chemistry, Tarbiat Modares University, P. O. Box 14155-175, Tehran, Iran.

[2] Department of Chemistry, Faculty of Basic Sciences, University of Birjand, P. O. Box 97175-615, Birjand, Iran.

* Corresponding author: Email: manaseri@birjand.ac.ir

General information

From the Merck Chemical Company, all chemicals were purchased. On a Bruker Avance DPX-250, NMR spectra were recorded using deuterated DMSO-d_6 and $CDCl_3$ as solvents and TMS as an internal standard.

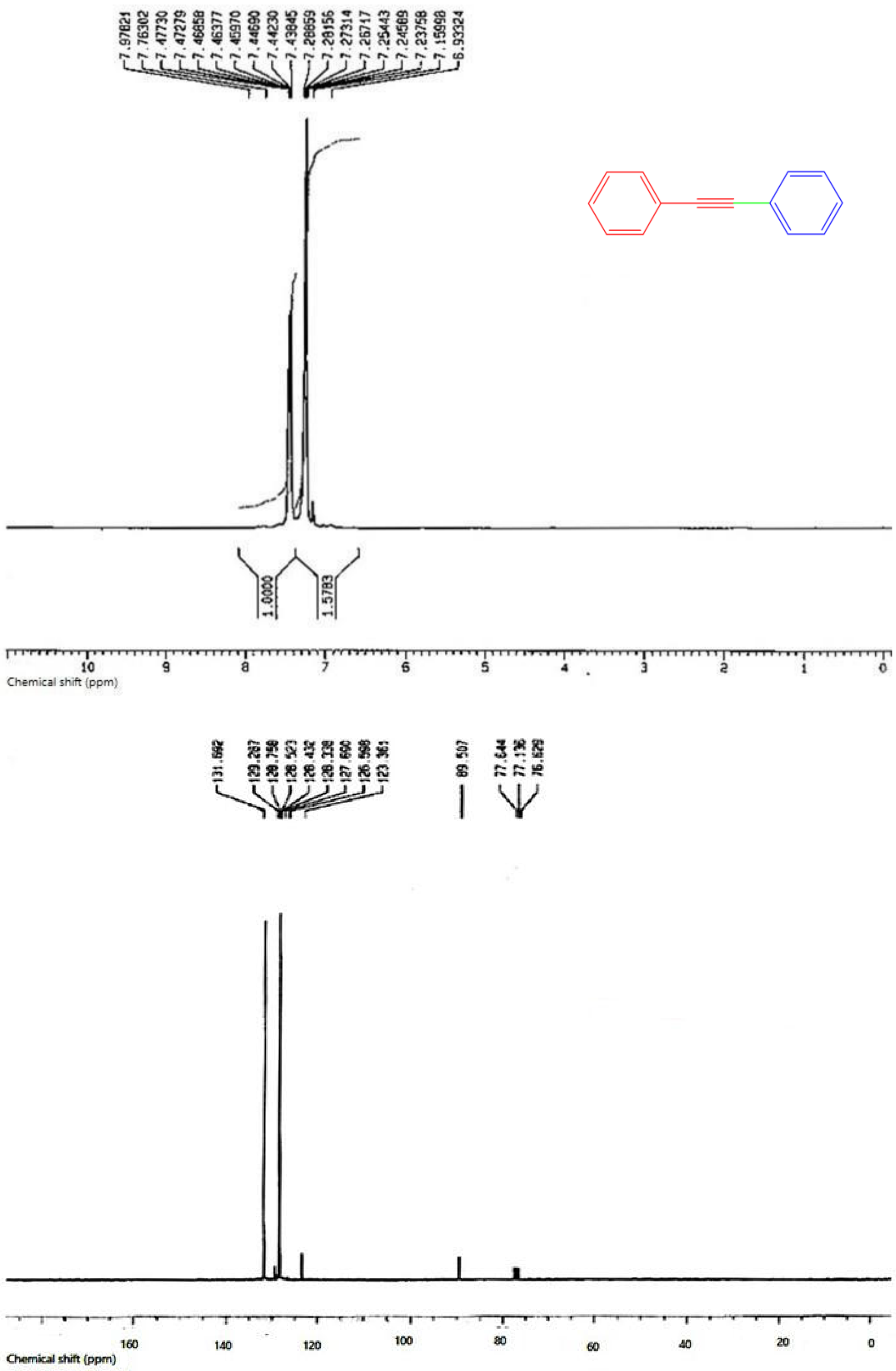

Figure S1. ^{1}H-NMR and ^{13}C-NMR spectra of Diphenylacetylene

Mp.: 61-63 °C (Lit.: 60-61 °C[1]); ^{1}H-NMR (250 MHz, $CDCl_3$); δ (ppm) = 7.44-7.98 (m, 5H), 6.93-7.29 (m, 5H); ^{13}C-NMR (62.5 MHz, $CDCl_3$): δ (ppm) = 131.5, 129.3, 128.8, 128.5, 128.3, 127.7, 126.5, 123.4, 89.5.

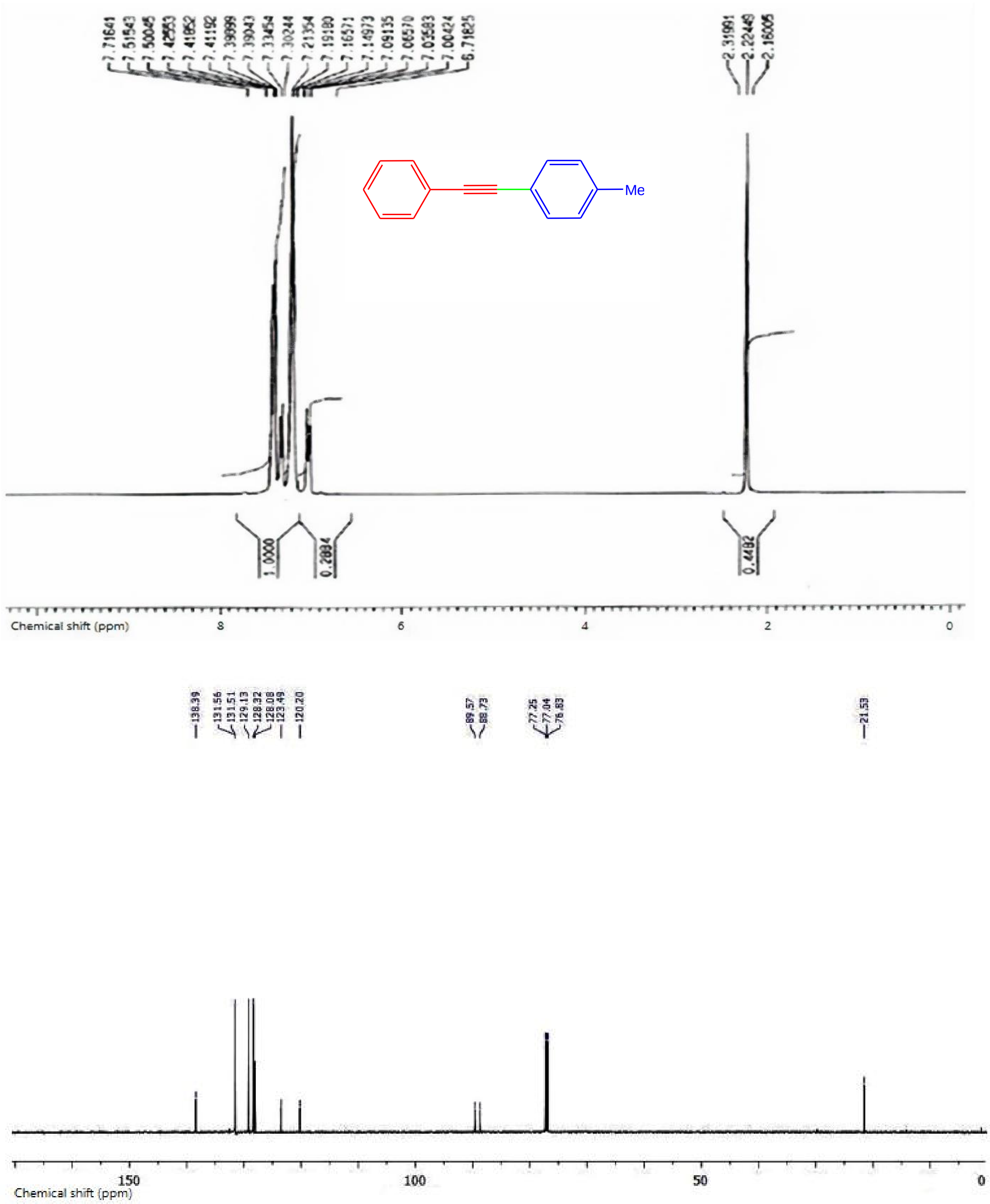

Figure S2. ^{1}H-NMR and ^{13}C-NMR spectra of 1-Methyl-4-(phenylethynyl)benzene

Mp.: 73-75 °C (Lit.: 72-74 °C[2]); ^{1}H-NMR (250 MHz, $CDCl_3$): δ (ppm) = 7.19–7.71 (m, 6H), 6.71-7.16 (m, 3H), 2.22 (s, 3H); ^{13}C-NMR (62.5 MHz, $CDCl_3$): δ (ppm) = 138.4, 131.6, 131.5, 129.1, 128.3, 128.0, 123.5, 120.2, 89.6, 88.7, 21.5.

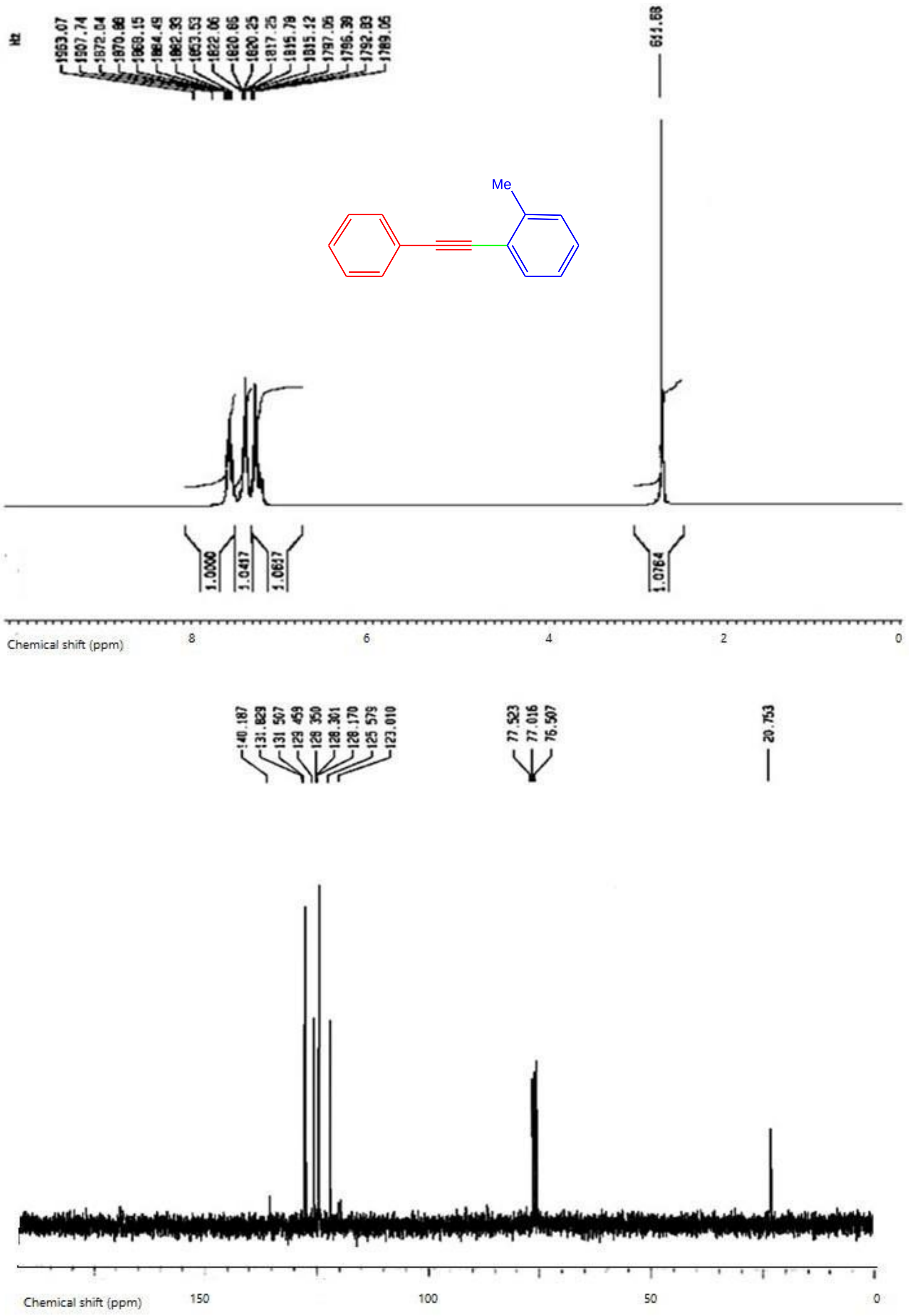

Figure S3. ^{1}H-NMR and ^{13}C-NMR spectra of 1-Methyl-2-(phenylethynyl)benzene

^{1}H-NMR (250 MHz, $CDCl_3$): δ (ppm) = 7.49–7.60 (m, 3H), 7.30–7.40 (m, 3H), 7.28-7.33 (m, 2H), 7.10–7.23 (m, 1H), 2.75 (s, 3H); ^{13}C-NMR (62.5 MHz, $CDCl_3$): δ (ppm) = 140.1, 132.5, 131.8, 131.5, 129.5, 129.3, 128.3, 128.2, 125.6, 123.0, 20.8.

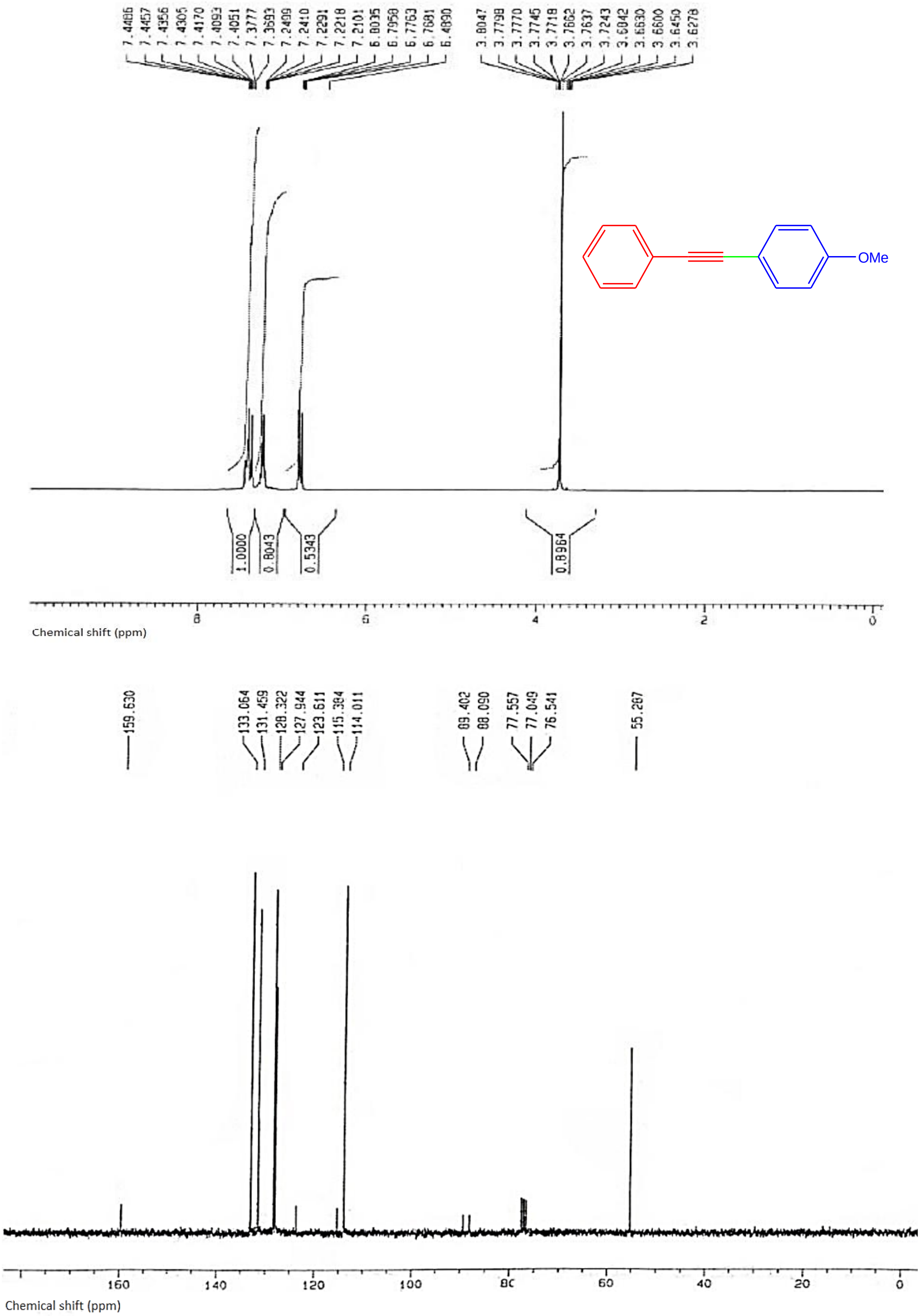

Figure S4. ^{1}H-NMR and ^{13}C-NMR spectra of 1-Methoxy-4-(phenylethynyl)benzene

Mp.: 57-59 °C (Lit.: 56-58 °C[2]); ^{1}H-NMR (250 MHz, $CDCl_3$) δ (ppm) = 7.37-7.44 (m, 4H), 7.21-7.25 (m, 3H), 6.79 (d, 2H, J= 8.2 Hz), 3.76 (s, 3H); ^{13}C-NMR (62.5 MHz, $CDCl_3$): δ (ppm) = 159.6, 133.0, 131.4, 128.3, 127.9, 123.6, 115.3, 114.0, 89.4, 88.0, 55.2.

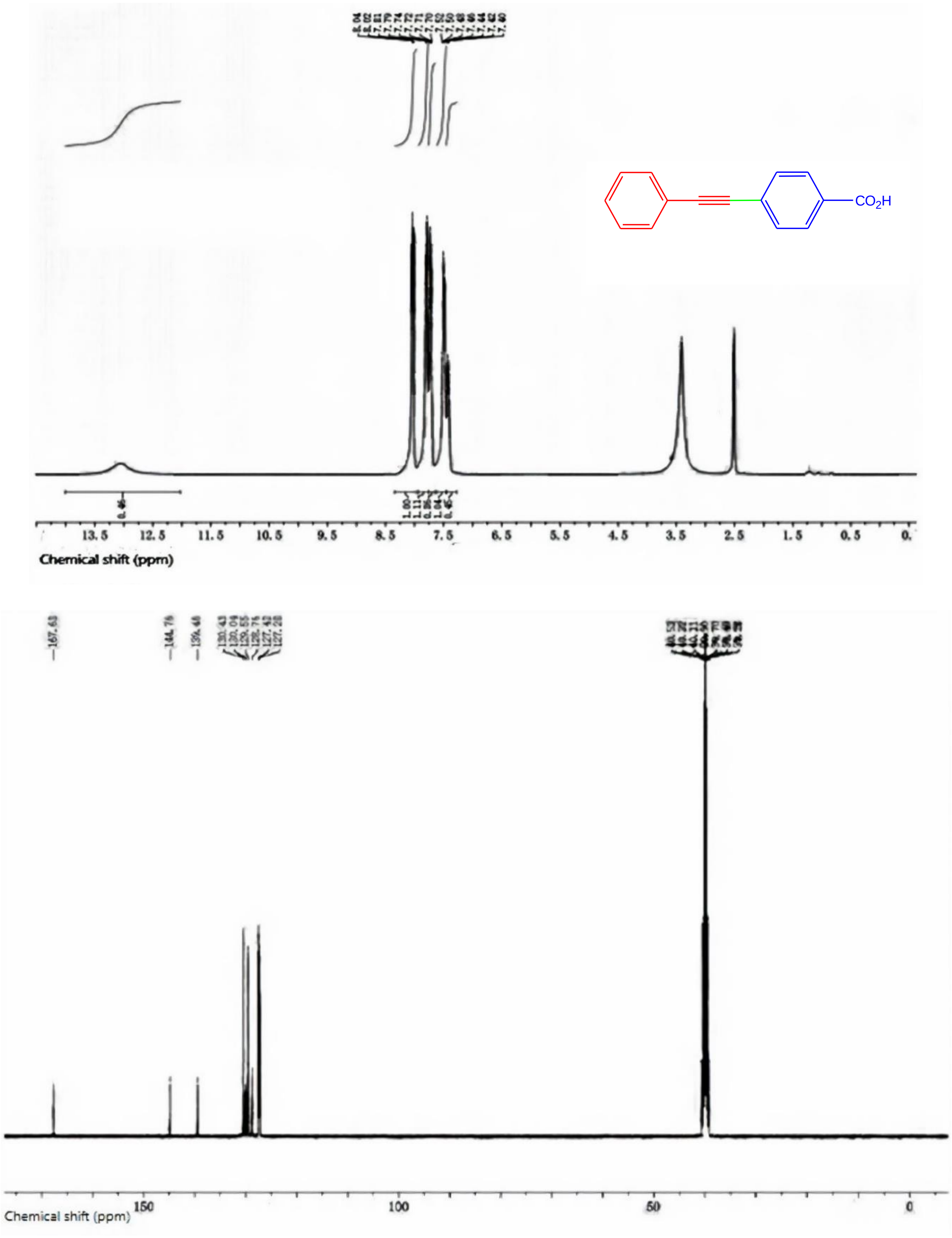

Figure S5. ^{1}H-NMR and ^{13}C-NMR spectra of 4-(Phenylethynyl)benzoic acid

Mp.: 223-225 °C (Lit.: 222-223 °C[3]); ^{1}H-NMR (250 MHz, DMSO-d_6): δ (ppm) = 12.98 (1H, s), 7.70-8.04 (6H, m), 7.49 (3H, m); ^{13}C-NMR (62.5 MHz, DMSO-d_6): δ (ppm) = 167.7, 145.1, 140.0, 130.5, 130.0, 129.6, 129.0, 127.5, 127.3.

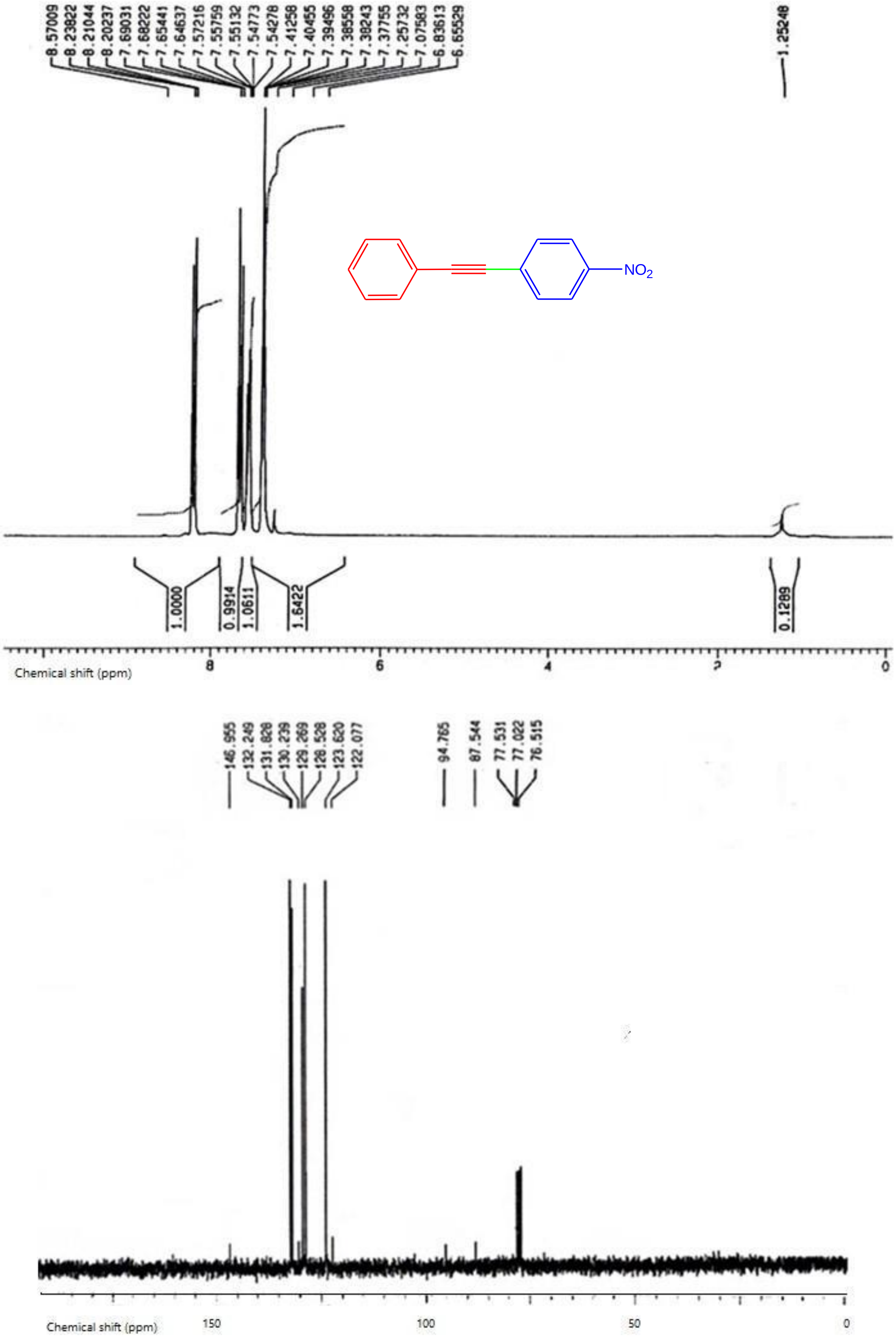

Figure S6. ^{1}H-NMR and ^{13}C-NMR spectra of 1-Nitro-4-(phenylethynyl)benzene

Mp.: 121-123 °C (Lit.: 120-122 °C[2]); ^{1}H-NMR (250 MHz, $CDCl_3$): δ (ppm) = 8.22 (d, *J*=7.7 Hz, 2H), 7.54-7.69 (m, 4H), 6.66-7.41 (m, 3H); ^{13}C-NMR (62.5 MHz, $CDCl_3$): δ (ppm) = 147.0, 132.3, 132.0, 130.2, 129.3, 128.5, 123.6, 122.0, 94.8, 87.5.

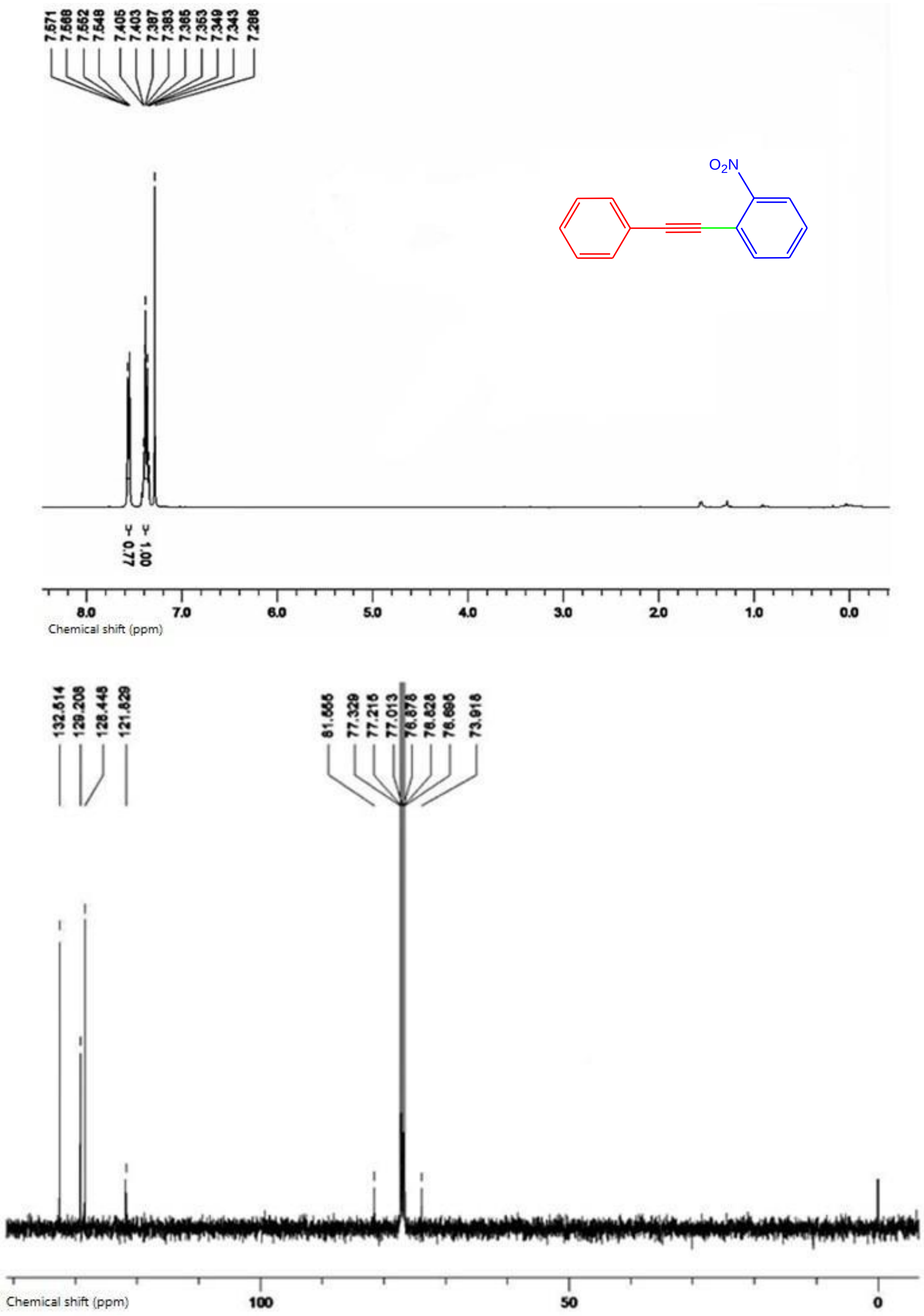

Figure S7. ^{1}H-NMR and ^{13}C-NMR spectra of 1-Nitro-2-(phenylethynyl)benzene

^{1}H-NMR (250 MHz, $CDCl_3$): δ (ppm) = 7.55-7.57 (m, 4H), 7.35-7.41 (m, 5H); ^{13}C-NMR (62.5 MHz, $CDCl_3$): δ (ppm) = 132.7, 129.2, 128.5, 121.7, 82.1, 74.0.

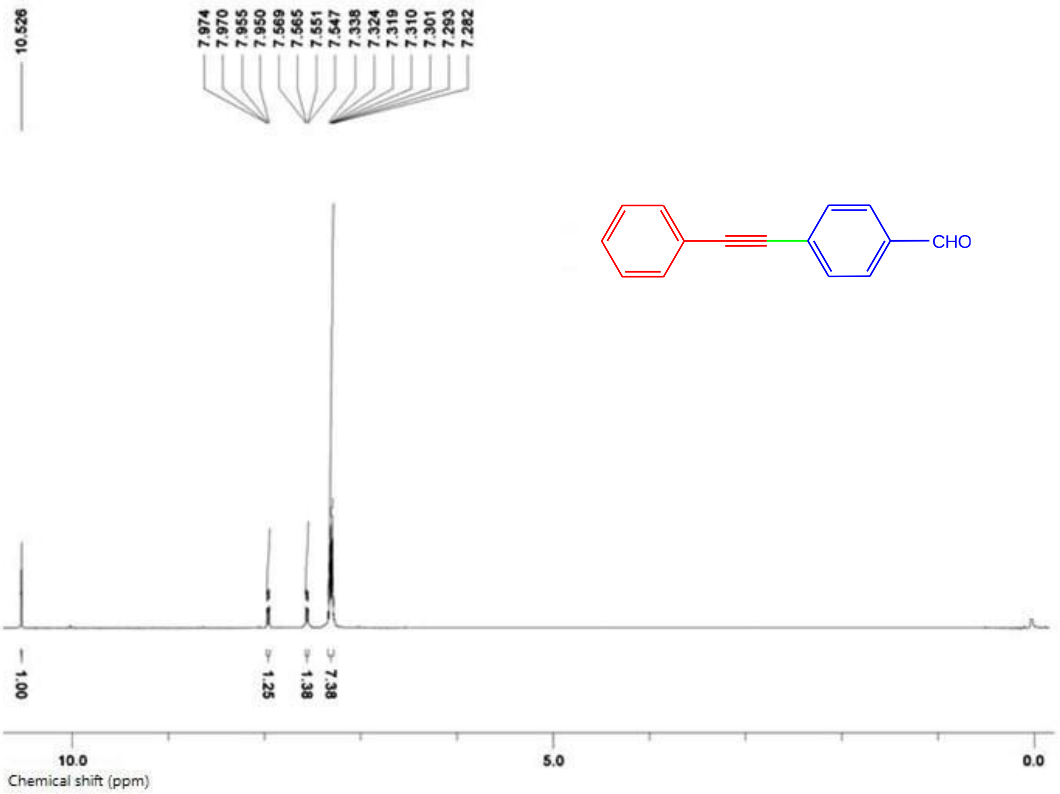

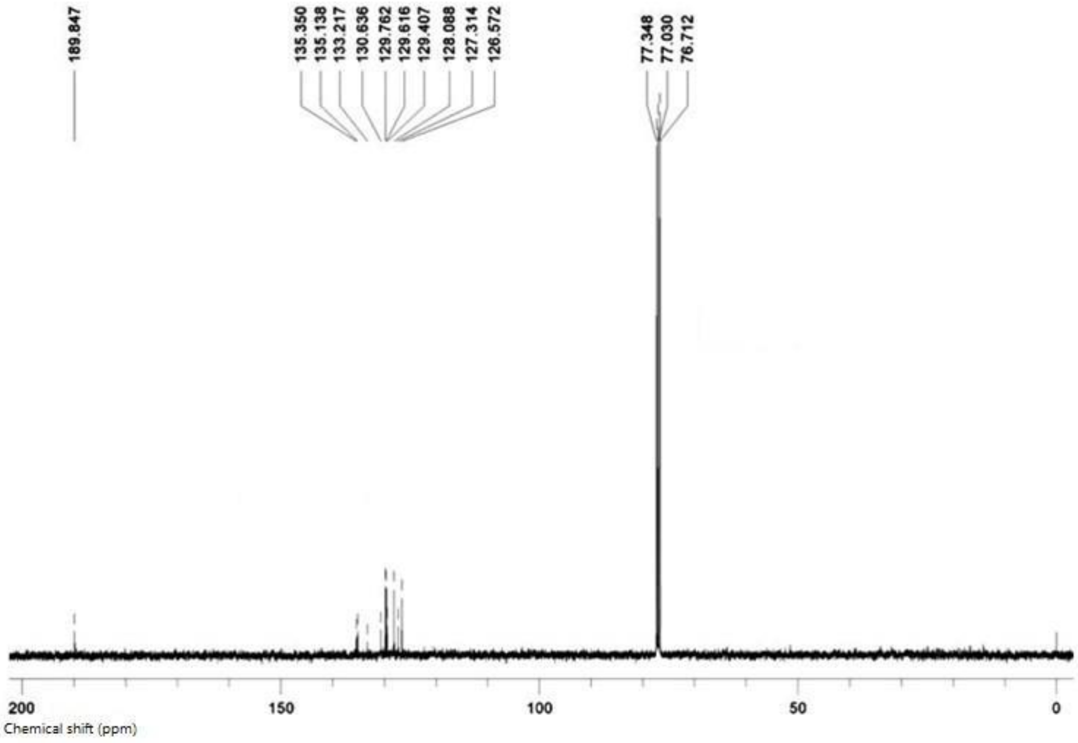

Figure S8. ^{1}H-NMR and ^{13}C-NMR spectra of 4-(Phenylethynyl)benzaldehyde

Mp.: 96-98 °C (Lit.: 95-96 °C[2]); ^{1}H-NMR (250 MHz, $CDCl_3$): δ (ppm) = 10.55 (s, 1 H), 7.96 (m, 1 H), 7.55 (m, 1 H), 7.28–7.34 (m, 7 H); ^{13}C-NMR (62.5 MHz, $CDCl_3$): δ (ppm) = 190.0, 135.4, 135.2, 133.3, 130.7, 129.8, 129.7, 129.4, 128.0, 127.3, 126.6.

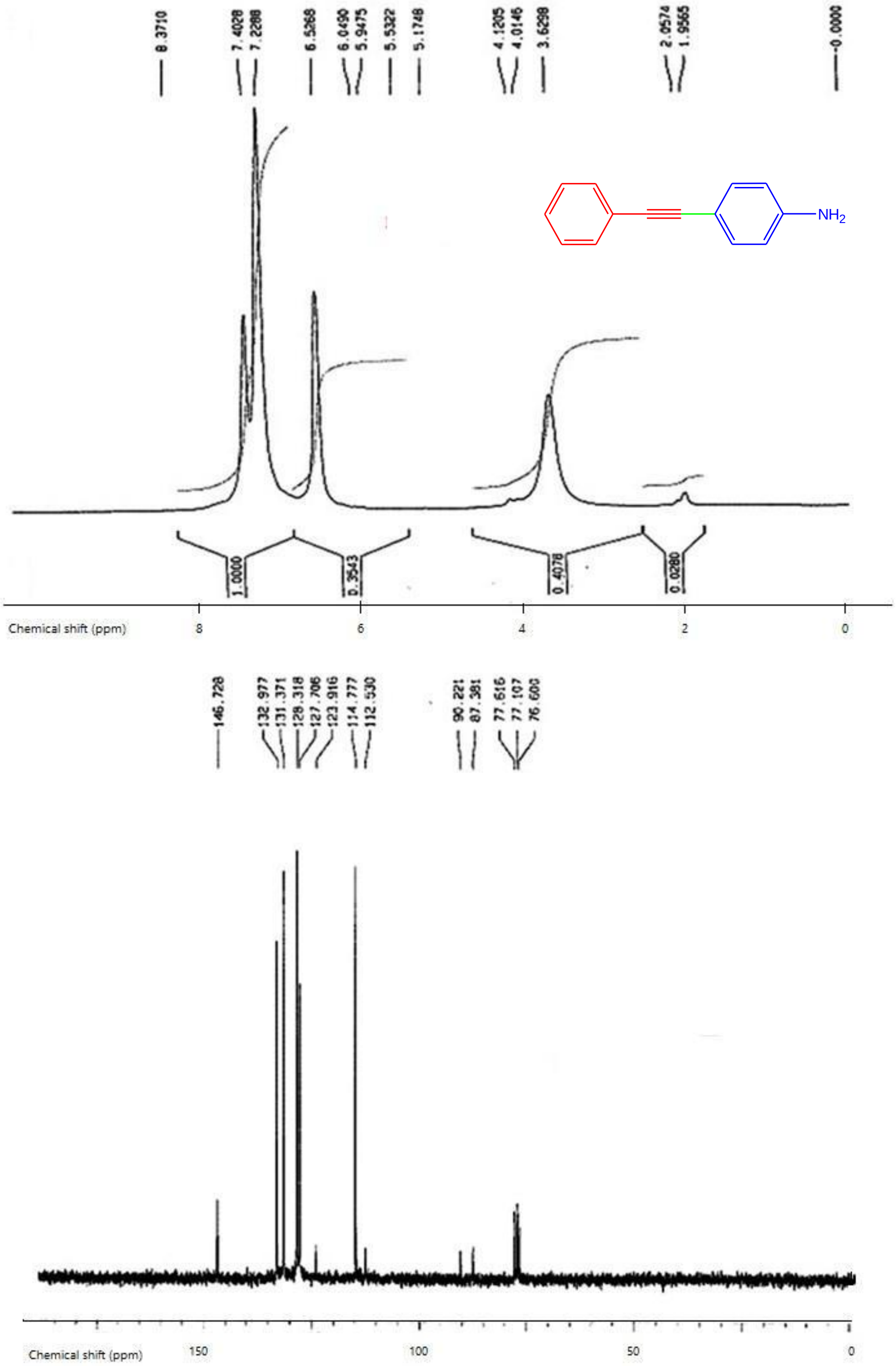

Figure S9. ^{1}H-NMR and ^{13}C-NMR spectra of 4-(Phenylethynyl)aniline

Mp.: 126-128 °C (Lit.: 125-126 °C[4]); ^{1}H-NMR (250 MHz, $CDCl_3$): δ (ppm) = 7.29-7.40 (m, 5H), 6.53 (m, 3H), 3.63 (s, 2H, NH_2). ^{13}C-NMR (62.5 MHz, $CDCl_3$): δ (ppm) = 146.8, 133.0, 131.4, 128.3, 127.8, 124.0, 115.0, 112.6, 90.2, 87.4.

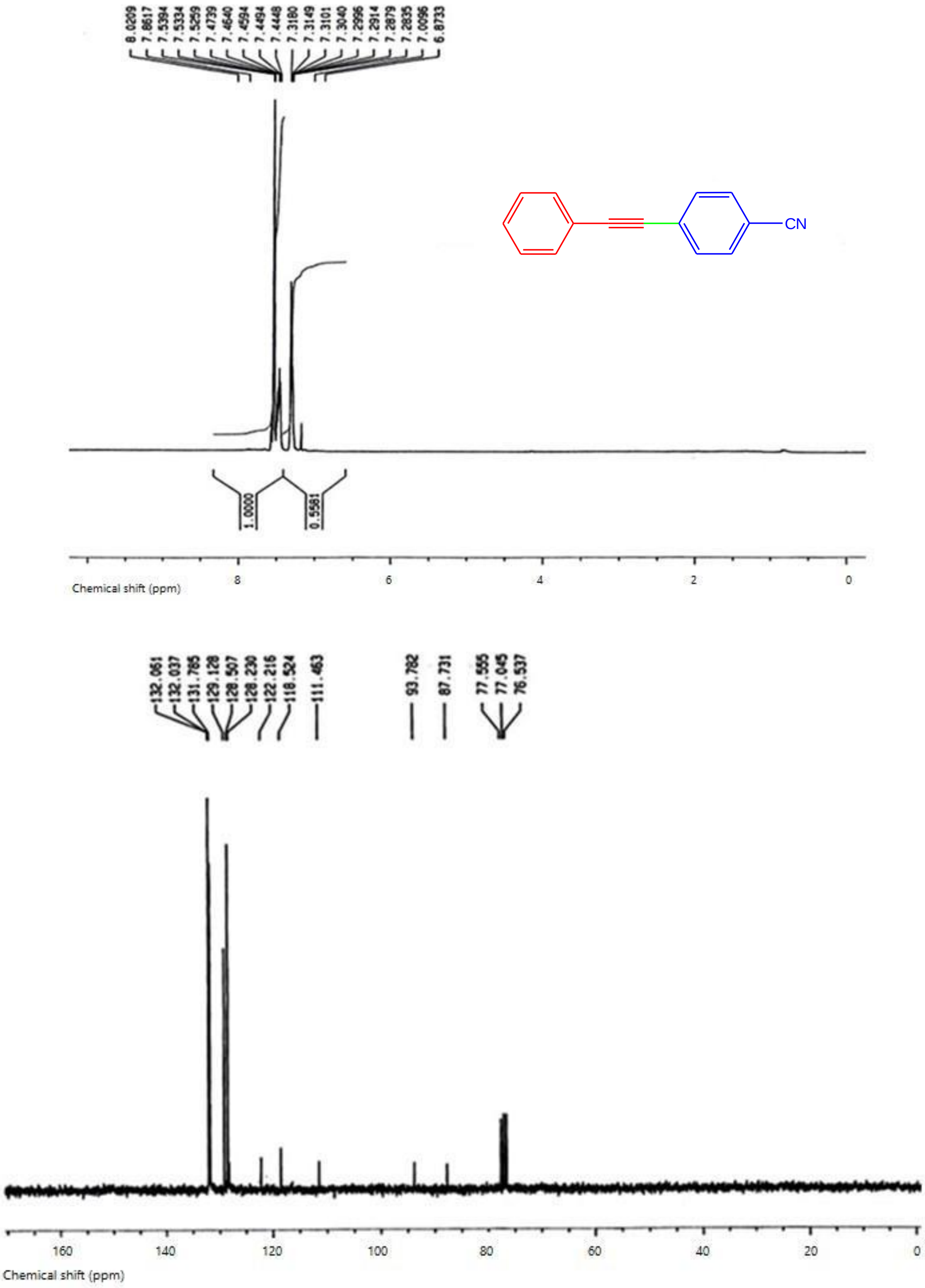

Figure S10. ^{1}H-NMR and ^{13}C-NMR spectra of 4-(Phenylethynyl)benzonitrile

Mp.: 104-107 °C (Lit.: 103-105 °C[5]); ^{1}H-NMR (250 MHz, $CDCl_3$): δ (ppm) = 7.45-7.54 (m, 6H), 7.28-7.31 (m, 3H); ^{13}C-NMR (62.5 MHz, $CDCl_3$): δ (ppm) = 132.5, 132.0, 130.7, 129.5, 129.0, 128.2, 122.2, 118.6, 111.4, 93.8, 87.7.

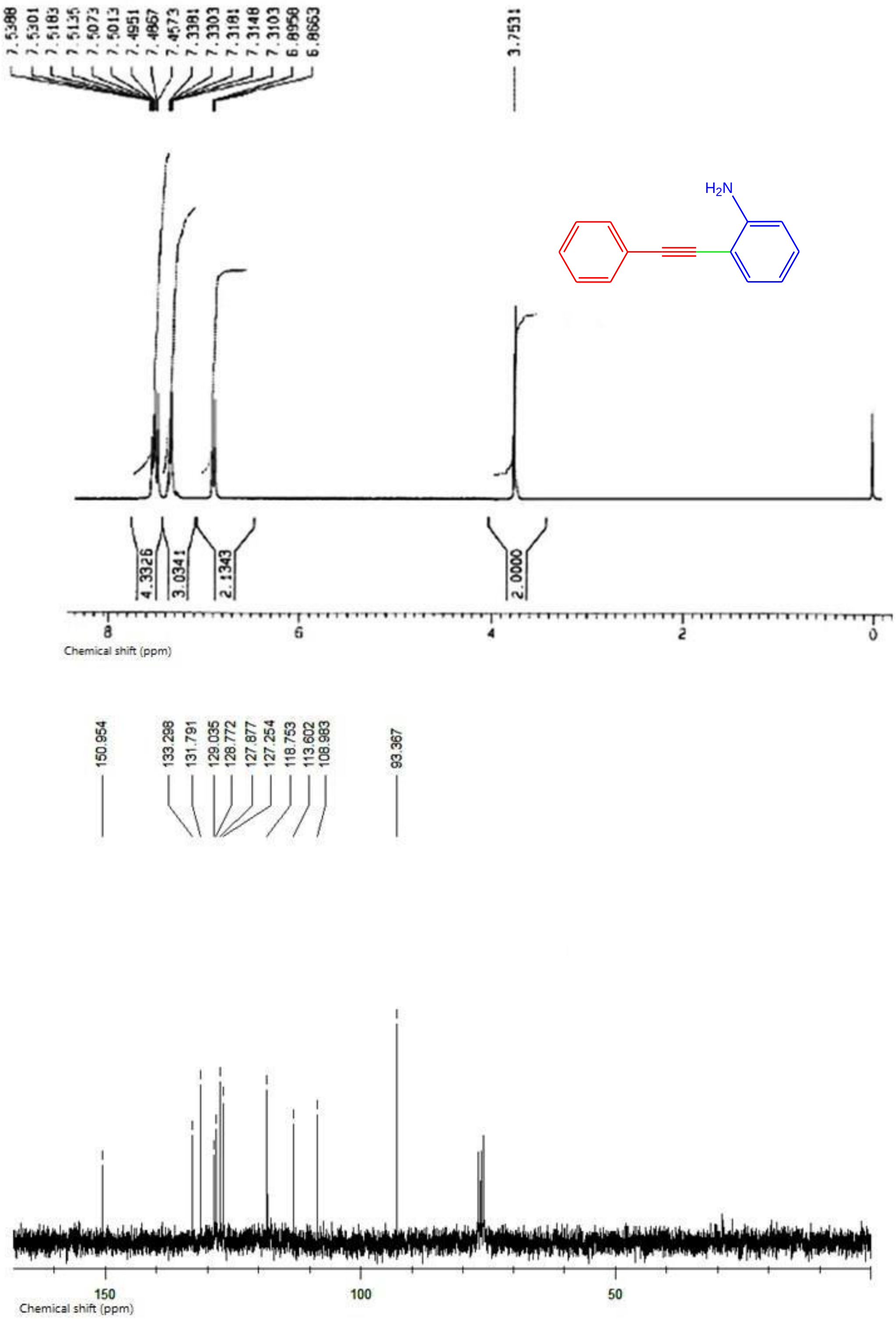

Figure S11. ^{1}H-NMR and ^{13}C-NMR spectra of 2-(Phenylethynyl)aniline

Mp.: 89-91 °C (Lit.: 87-88 °C[6]); ^{1}H-NMR (250 MHz, $CDCl_3$): δ (ppm) = 7.46 - 7.54 (m, 4 H), 7.31-7.34 (m, 3 H), 6.75 (m, 2 H), 3.76 (s, 2 H); ^{13}C-NMR (62.5 MHz, $CDCl_3$): δ (ppm) = 151.0, 133.3, 131.8, 129.0, 128.8, 127.9, 127.3, 118.8, 113.6, 108.0, 93.4.

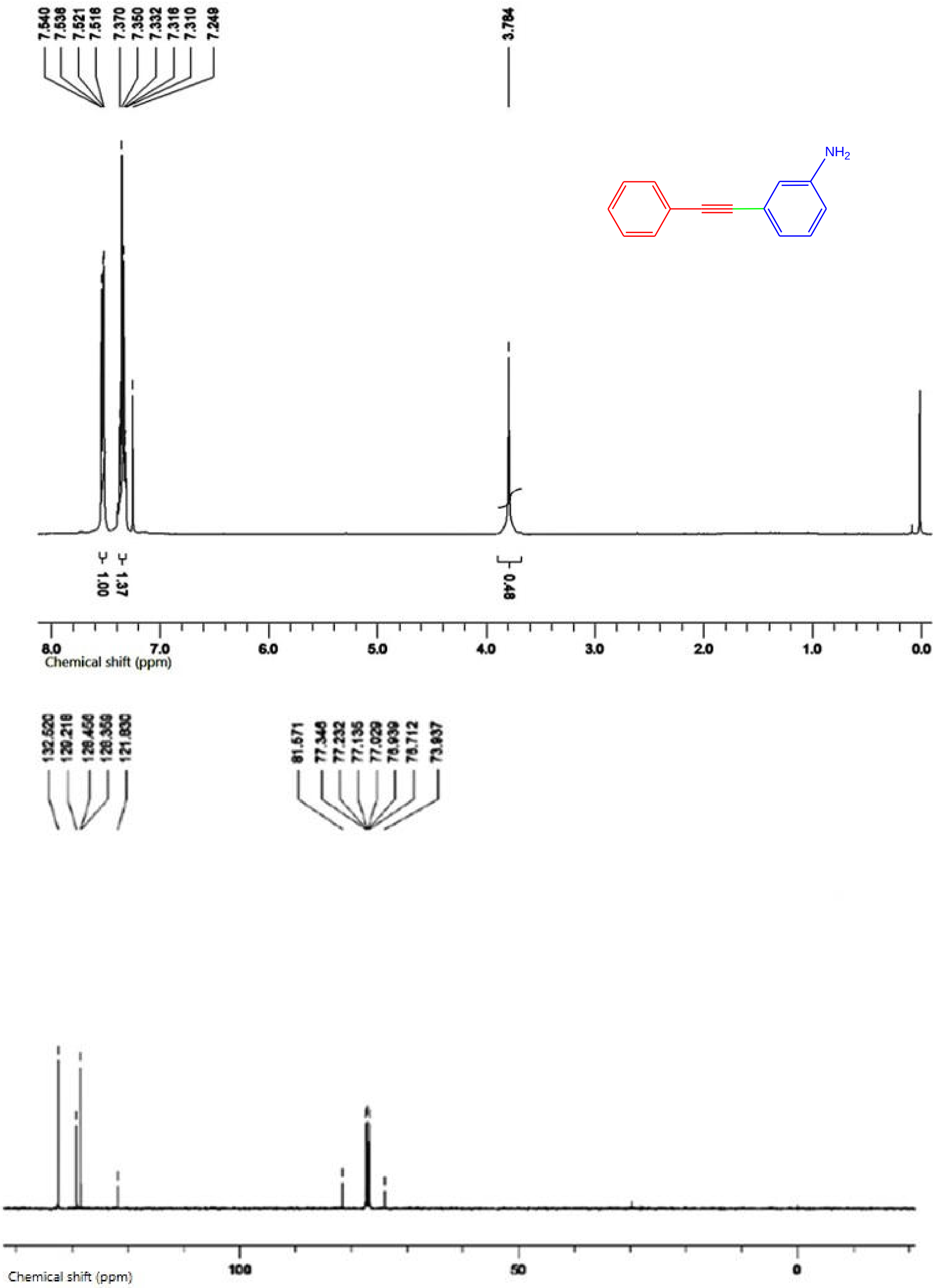

Figure S12. ^{1}H-NMR and ^{13}C-NMR spectra of 3-(Phenylethynyl)aniline

^{1}H-NMR (250 MHz, $CDCl_3$): δ (ppm) = 7.52-7.54 (m, 4H), 7.25-7.37 (m, 6H), 3.79 (s, 2H). ^{13}C-NMR (62.5 MHz, $CDCl_3$): δ (ppm) = 132.6, 129.3, 128.5, 128.4, 121.9, 81.7, 74.0.

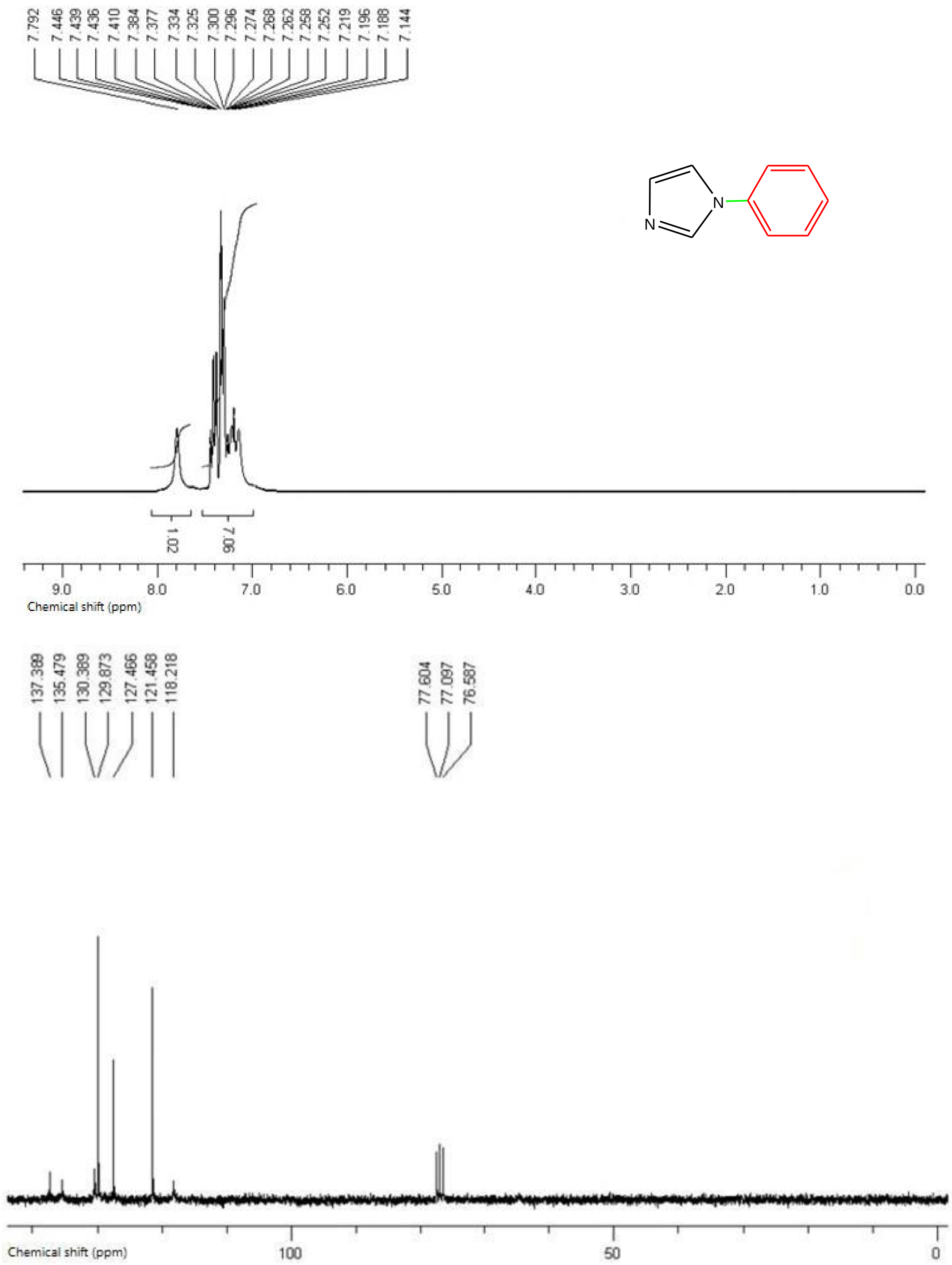

Figure S13. ^{1}H-NMR and ^{13}C-NMR spectra of 1-Phenyl-1*H*-imidazole

^{1}H-NMR (250 MHz, $CDCl_3$): δ (ppm) = 7.80 (s, 1H), 7.15-7.45 (m, 6H); ^{13}C-NMR (62.5 MHz, $CDCl_3$): δ (ppm) = 137.4, 135.5, 130.3, 130.0, 127.5, 121.5, 118.2.

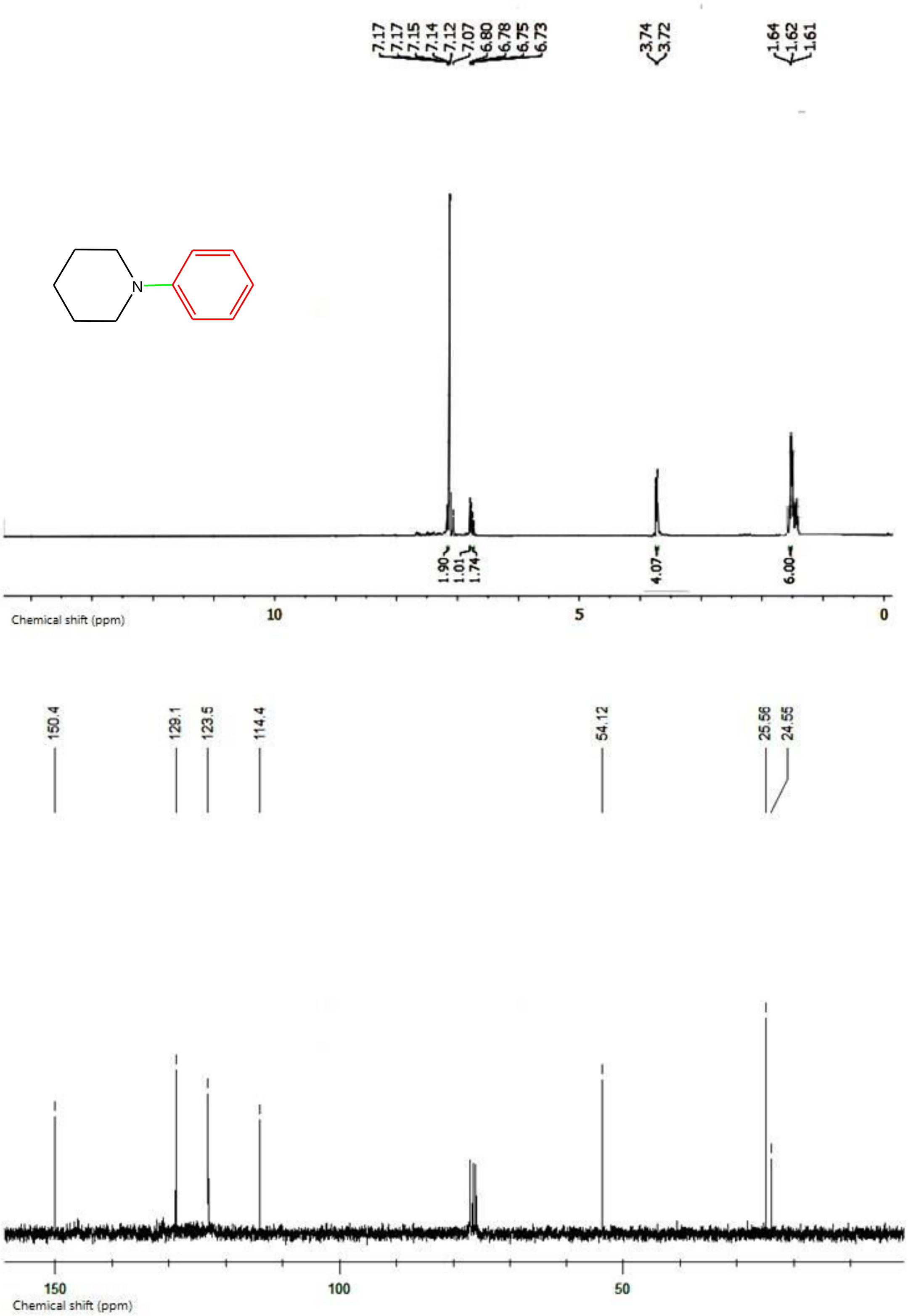

Figure S14. ^{1}H-NMR and ^{13}C-NMR spectra of 1-Phenylpiperidine

^{1}H-NMR (250 MHz, $CDCl_3$): δ (ppm) = 7.22-7.25 (m, 2H), 7.07-7.17 (m, 2H), 6.93 (m, 2H), 6.73-6.80 (m, 3H), 3.73 (d, J=5.0 Hz, 4H), 1.62 (m, 6H); ^{13}C-NMR (62.5 MHz, $CDCl_3$): δ (ppm) = 150.5, 129.1, 123.6, 114.4, 54.1, 25.6, 24.6.

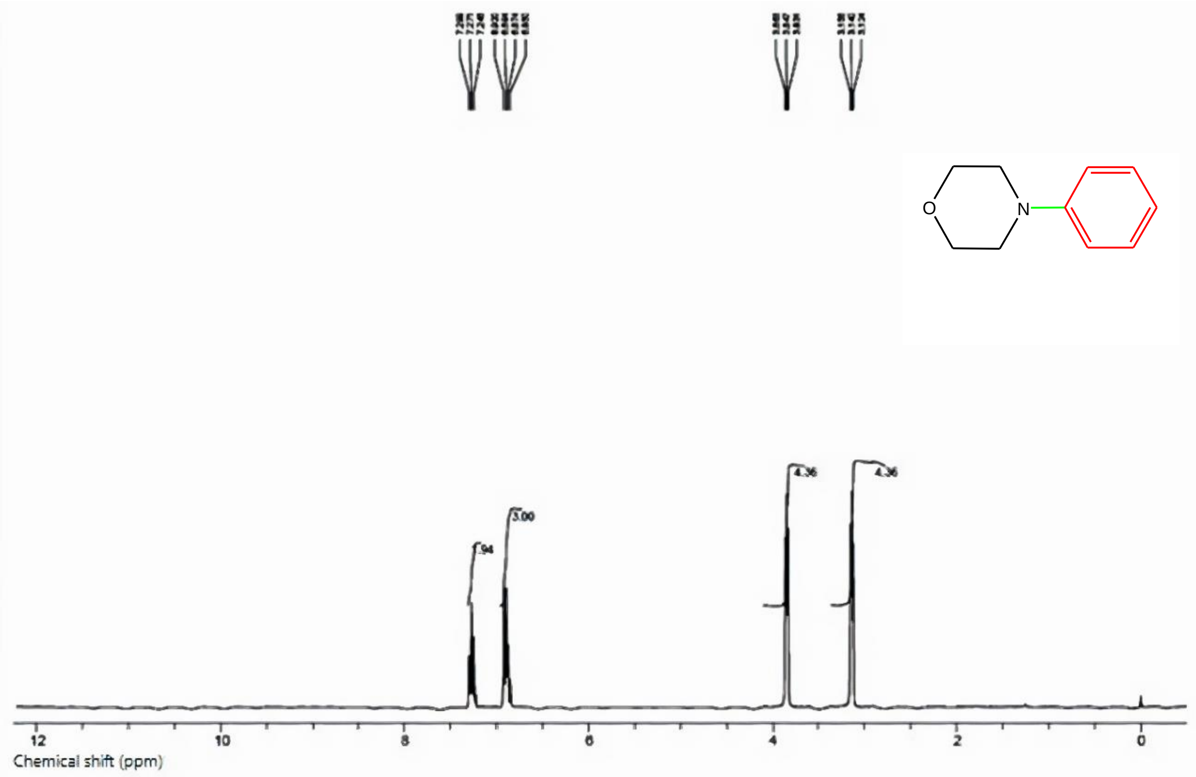

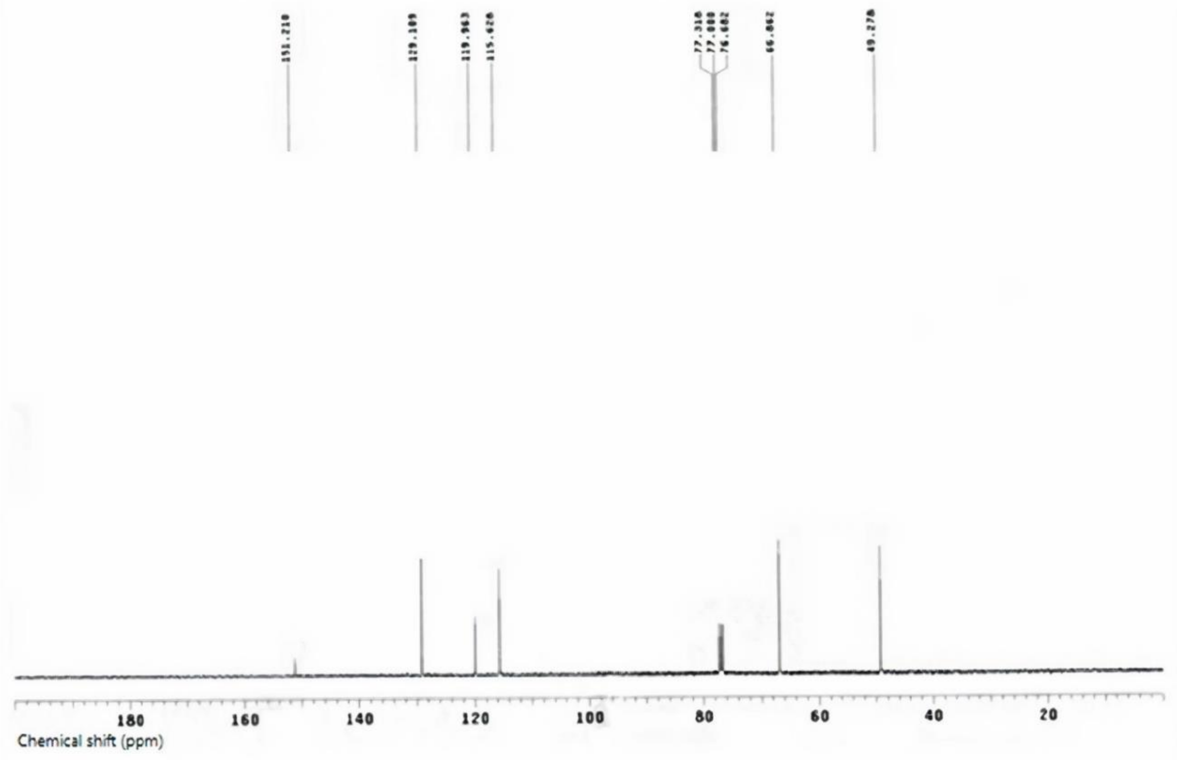

Figure S15. ^{1}H-NMR and ^{13}C-NMR spectra of 4-Phenylmorpholine

Mp.: 54-56 °C (Lit.: 53–54 °C[7]); ^{1}H-NMR (250 MHz, $CDCl_3$): δ (ppm) = 7.27 (m, 2H), 6.60 (m, 3H), 3.30 (t, J_1=4.0 Hz, J_2=7.5 Hz, 4H), 3.14 (t, J_1=4.0 Hz, J_2=7.5 Hz, 4H); ^{13}C-NMR (62.5 MHz, $CDCl_3$): δ (ppm) = 151.2, 129.1, 119.9, 115.6, 66.8, 49.2.

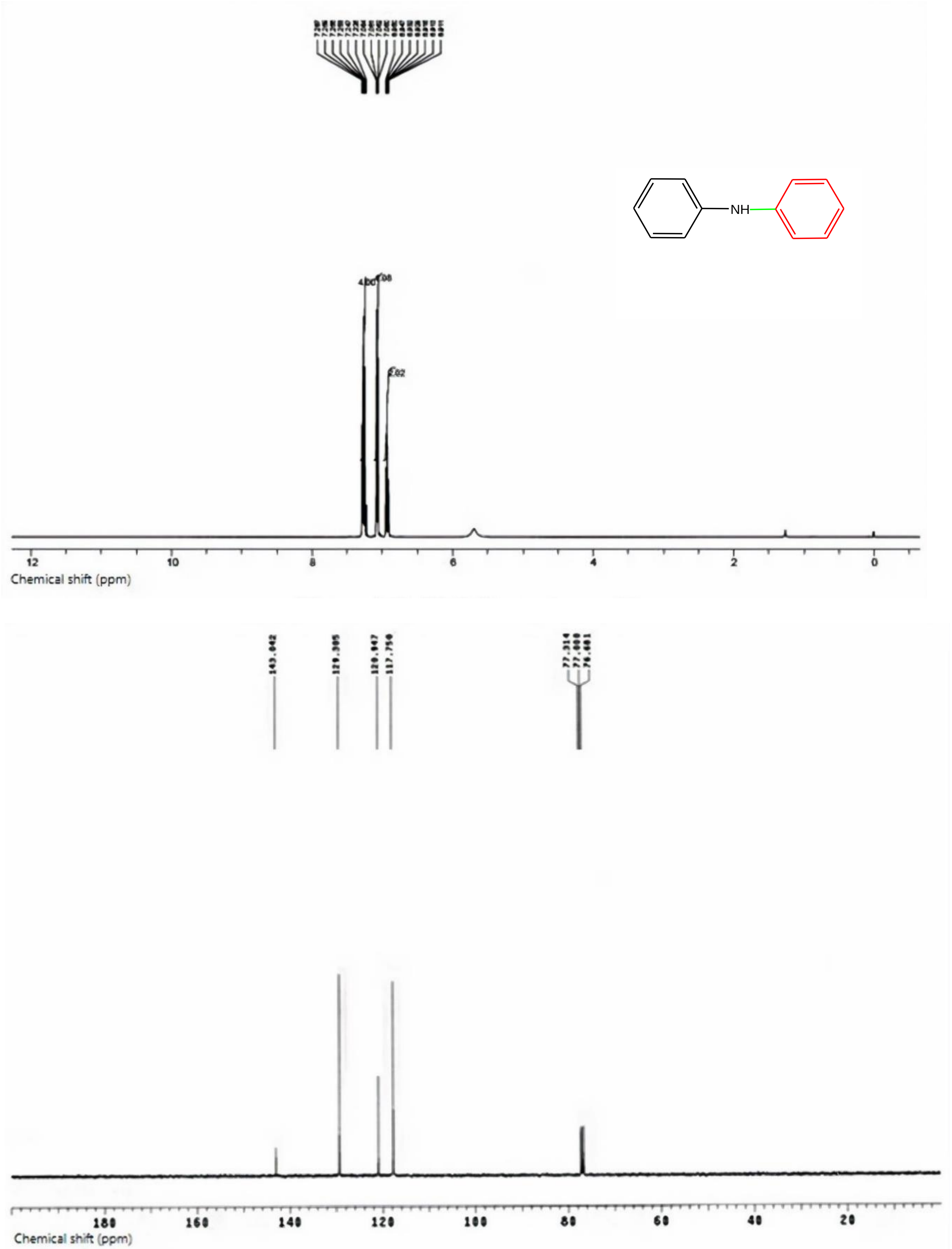

Figure S16. ^{1}H-NMR and ^{13}C-NMR spectra of Diphenylamine

Mp.: 53-55 °C (Lit.: 51–52 °C[8]); ^{1}H-NMR (250 MHz, $CDCl_3$): δ (ppm) = 7.26 (m, 4H), 7.10 (m, 4H), 6.95 (m, 2H), 5.64 (s, br, 1H); ^{13}C-NMR (62.5 MHz, $CDCl_3$): δ (ppm) = 145.9, 130.0, 121.0, 117.8.

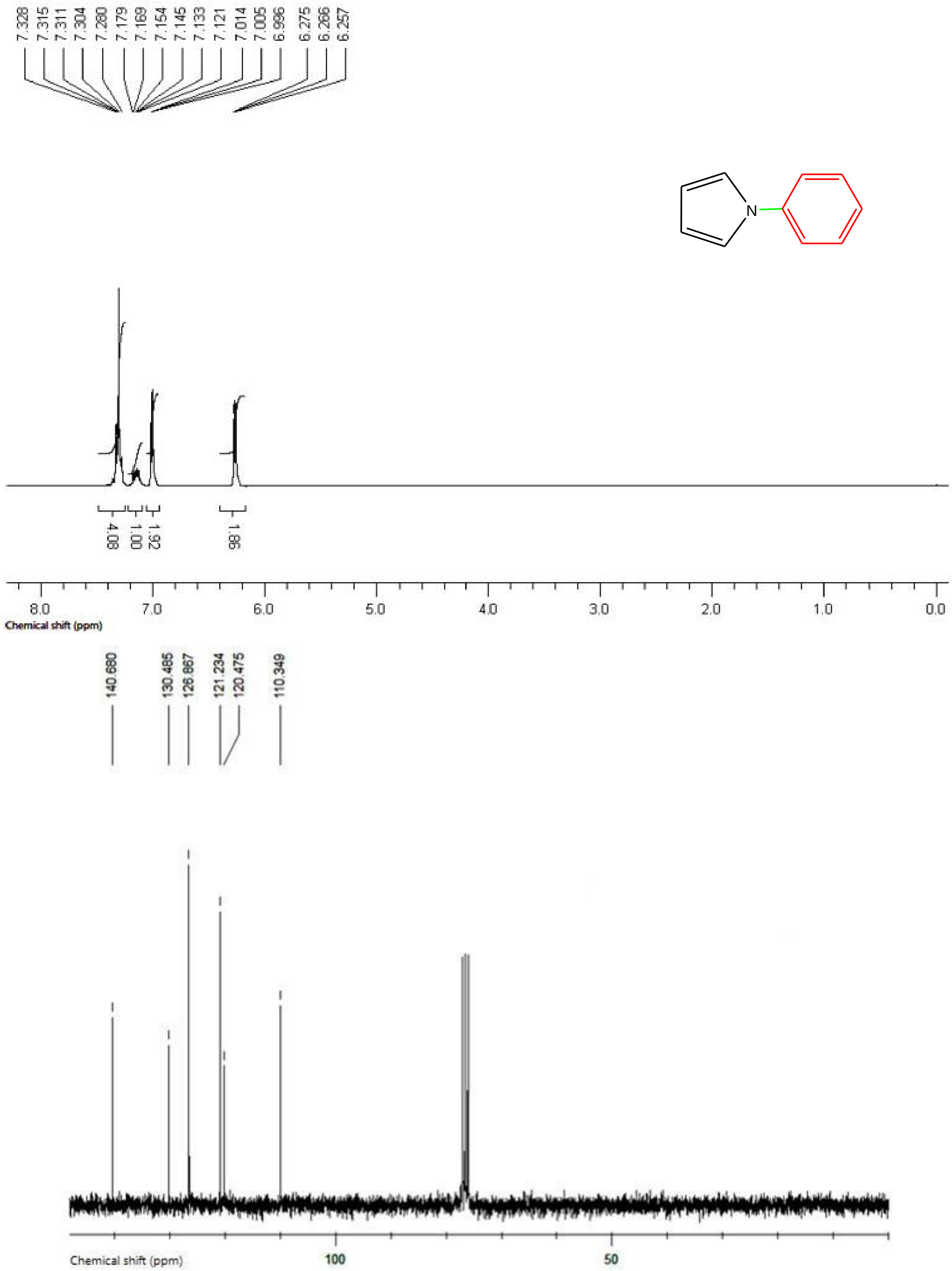

Figure S17. ^{1}H-NMR and ^{13}C-NMR spectra of 1-Phenyl-1*H*-pyrrole

^{1}H-NMR (250 MHz, $CDCl_3$): δ (ppm) = 7.28-7.33 (m, 4H), 7.12-7.18 (m, 1H), 7.00 (m, 2H), 6.27 (t, J_1=2.25 Hz, J_2=4.5 Hz, 2H); ^{13}C-NMR (62.5 MHz, $CDCl_3$): δ (ppm) = 140.7, 130.5, 126.9, 121.2, 120.5, 110.4.

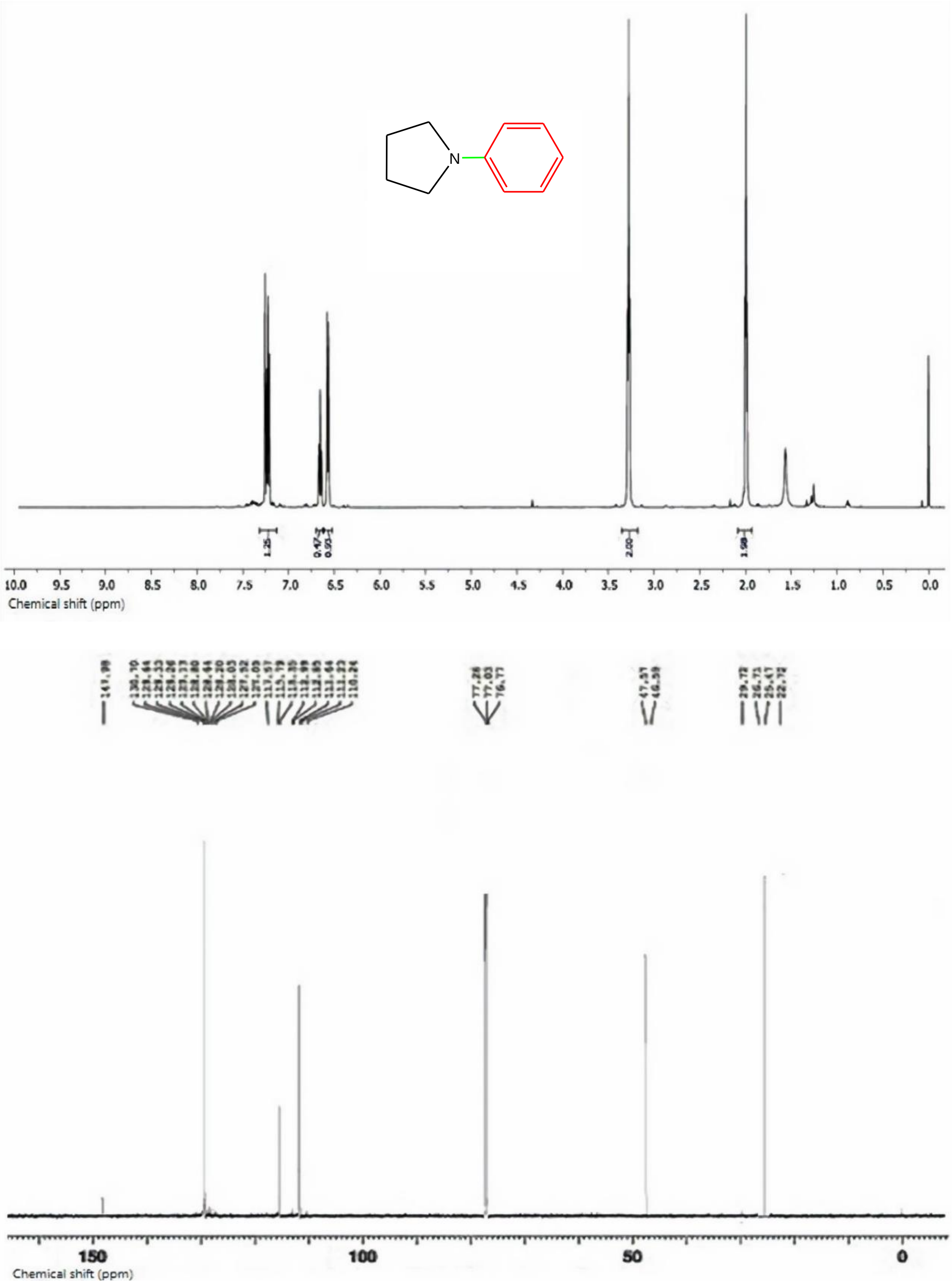

Figure S18. ^{1}H-NMR and ^{13}C-NMR spectra of 1-Phenylpyrrolidine

^{1}H-NMR (250 MHz, $CDCl_3$): δ (ppm) = 7.20 (m, 2H), 6.55-6.60 (m, 3H), 3.27 (t, J_1= 5.0 Hz, J_2=7.5 Hz, 4H), 2.05 (t, J_1= 5.0 Hz, J_2=7.5 Hz, 4H); ^{13}C-NMR (62.5 MHz, $CDCl_3$): δ (ppm) = 150.7, 130.0, 121.8, 114.7, 51.3, 25.3.

Mp.: 208-210 °C (Lit.: 208–209 °C[9]); ^{1}H-NMR (250 MHz, $CDCl_3$): δ (ppm) = 8.30 (m, 2H), 7.93 (s, 1 H), 7.52 (d, *J*=7.5 Hz, 2 H), 7.32 (d, *J*=7.7 Hz, 1 H), 7.21 (d, *J*=7.7 Hz, 1 H); ^{13}C-NMR (62.5 MHz, $CDCl_3$): δ (ppm) = 147.9, 143.1, 136.2, 130.2, 125.0, 122.0, 118.6.

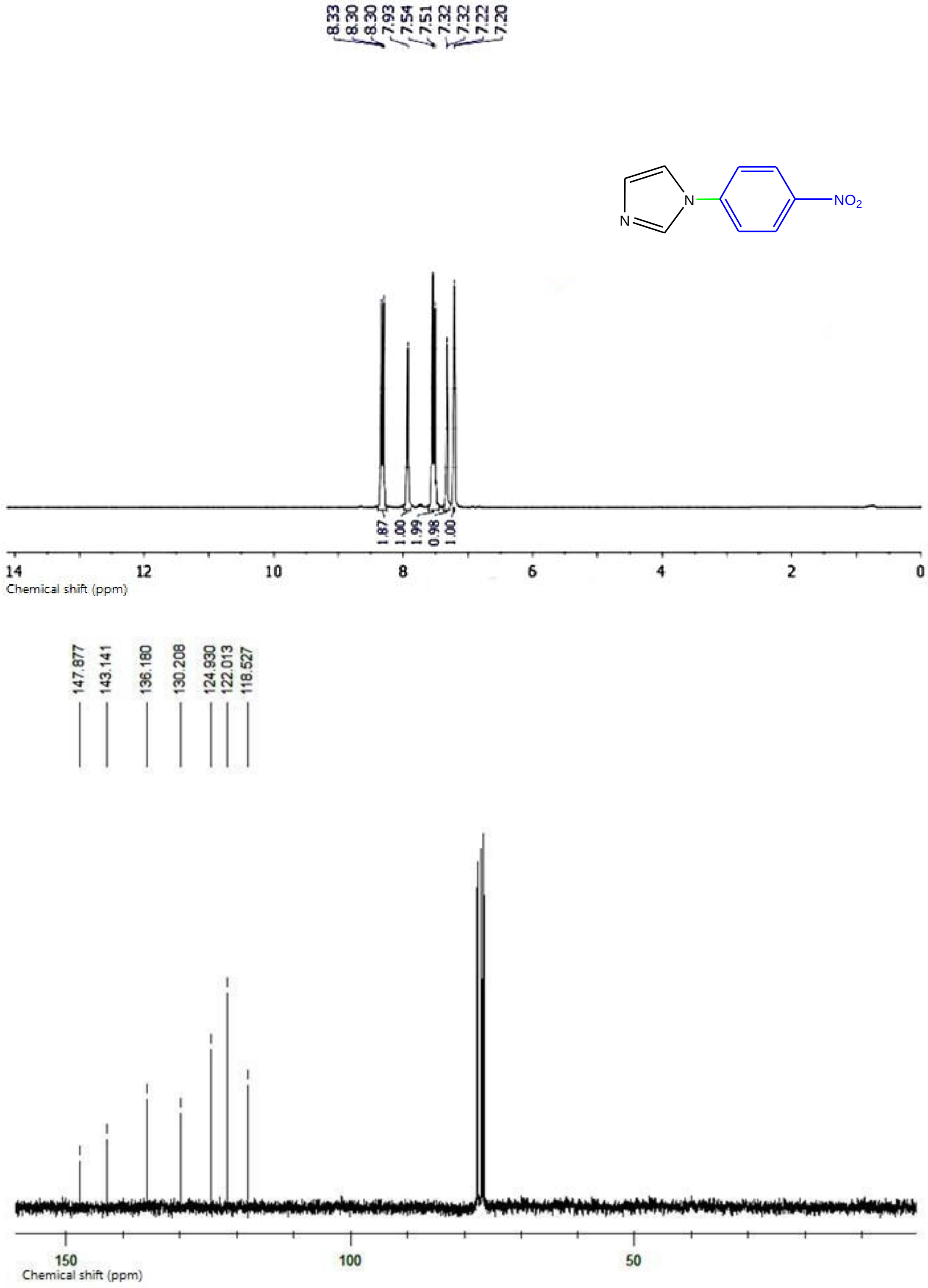

Figure S19. ^{1}H-NMR and ^{13}C-NMR spectra of 1-(4-Nitrophenyl)-1*H*-imidazole

The first line reads 20.5.

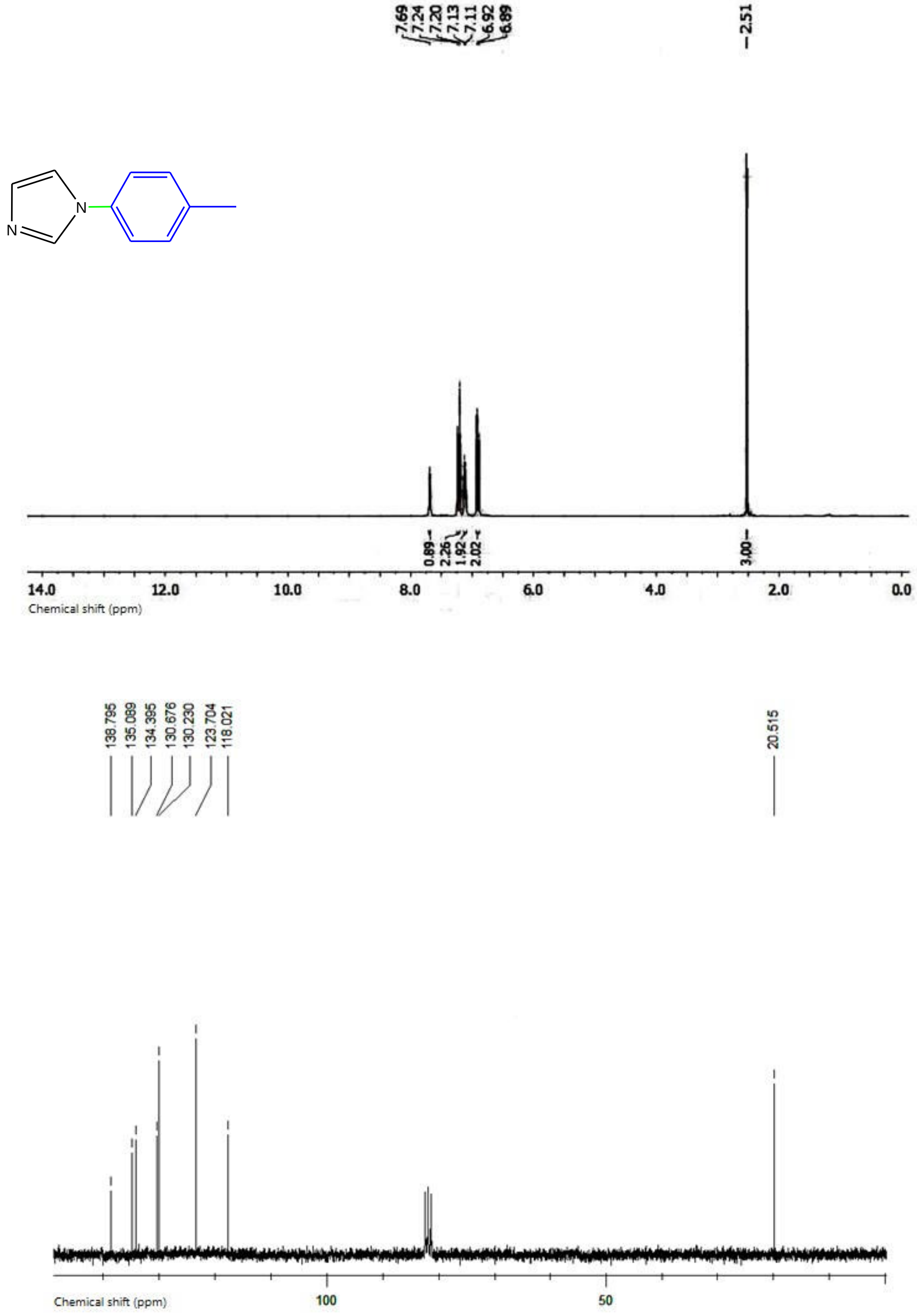

Figure S20. ^{1}H-NMR and ^{13}C-NMR spectra of 1-(p-Tolyl)-1*H*-imidazole

Mp.: 36-38 °C (Lit.: 35 °C[10]); ^{1}H-NMR (250 MHz, $CDCl_3$): δ (ppm) = 7.70 (s, 1H), 7.22 (d, *J*=7.5 Hz, 2H), 7.12 (d, *J*=7.5 Hz, 2H), 6.90 (d, *J*=7.5 Hz, 2H), 2.51 (s, 3H); ^{13}C-NMR (62.5 MHz, $CDCl_3$): δ (ppm) = 138.8, 135.1, 134.4, 130.7, 130.2, 123.7, 118.0, 20.5.

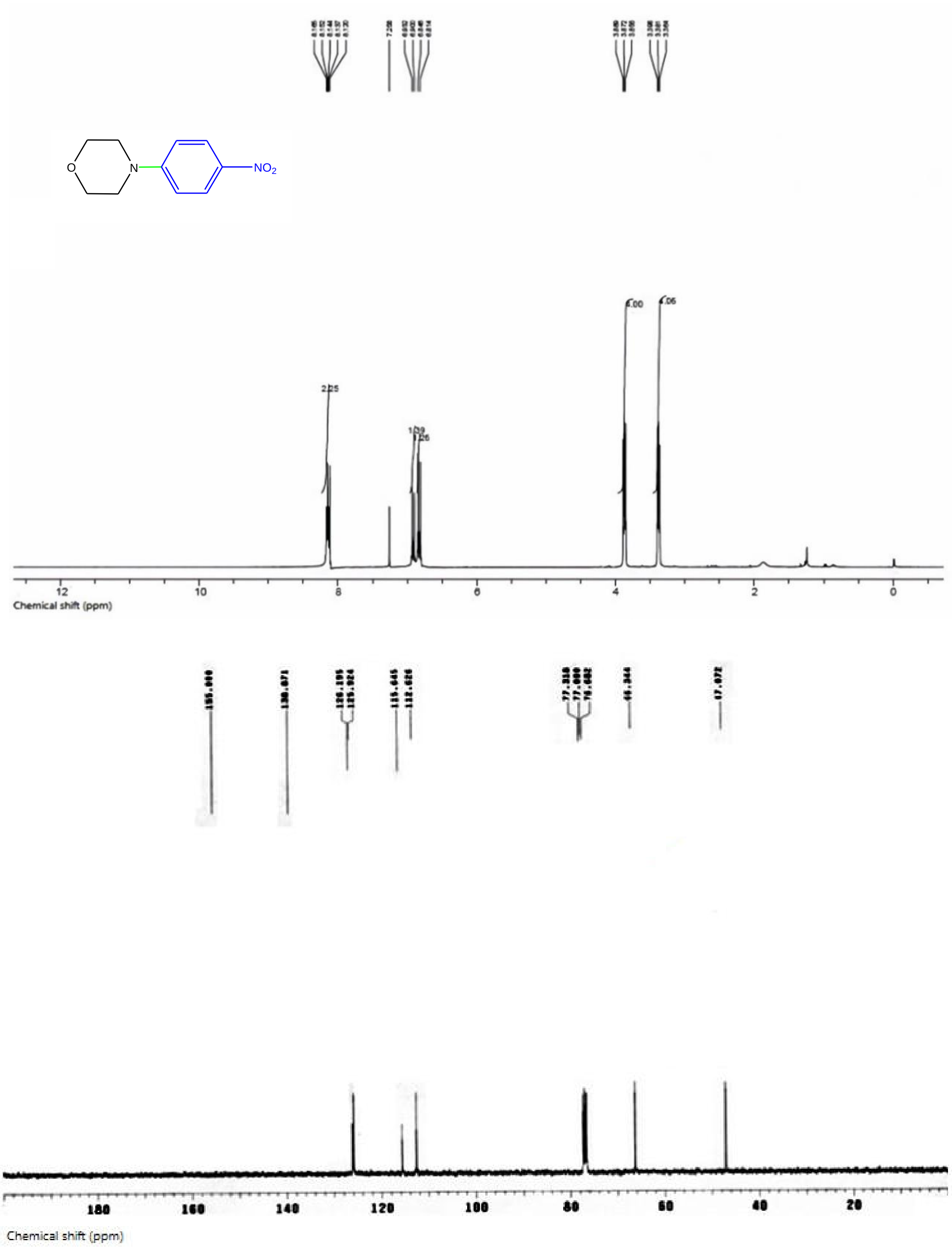

Figure S21. ^{1}H-NMR and ^{13}C-NMR spectra of 4-(4-Nitrophenyl)morpholine

Mp.: 154-158 °C (Lit.: 153-156 °C[8]); ^{1}H-NMR (250 MHz, $CDCl_3$): δ (ppm) = 8.14 (m, 2H), 7.20 (m, 1H), 6.80 (m, 1H), 3.47 (t, J_1=4.25 Hz, J_2=7.5 Hz, 4H), 3.38 (t, J_1=4.25 Hz, J_2=7.5 Hz, 4H); ^{13}C-NMR (62.5 MHz, $CDCl_3$): δ (ppm) = 154.2, 136.9, 124.3, 112.6, 66.5, 54.3.

References

1 Novák, Z., Nemes, P. & Kotschy, A. Tandem Sonogashira coupling: An efficient tool for the synthesis of diarylalkynes. *Org. Lett.* **6**, 4917-4920 (2004). https://doi.org:10.1021/ol047983f

2 Planellas, M. *et al.* Heck, Sonogashira, and Hiyama reactions catalyzed by palladium nanoparticles stabilized by tris-imidazolium salt. *Eur. J. Org. Chem.* **2014**, 3001-3008 (2014).

3 Zhang, X. *et al.* Silver(i)-catalyzed carboxylation of arylboronic esters with CO_2. *ChemComm.* **48**, 6292-6294 (2012). https://doi.org:10.1039/C2CC32045B

4 Gogoi, A., Dewan, A., Borah, G. & Bora, U. A palladium salen complex: An efficient catalyst for the Sonogashira reaction at room temperature. *New J. Chem.* **39**, 3341-3344 (2015). https://doi.org:10.1039/C4NJ01822B

5 Edwin Raja, G. C., Irudayanathan, F. M., Kim, H.-S., Kim, J. & Lee, S. Nickel-catalyzed Hiyama-type decarboxylative coupling of propiolic acids and organosilanes. *J. Org. Chem.* **81**, 5244-5249 (2016). https://doi.org:10.1021/acs.joc.6b00883

6 Zille, M., Stolle, A., Wild, A. & Schubert, U. S. $ZnBr_2$-mediated synthesis of indoles in a ball mill by intramolecular hydroamination of 2-alkynylanilines. *RSC Adv.* **4**, 13126-13133 (2014).

7 Zhang, Z. *et al.* Highly efficient and practical phosphoramidite–copper catalysts for amination of aryl iodides and heteroaryl bromides with alkylamines and N (H)-heterocycles. *Tetrahedron.* **62**, 4435-4443 (2006).

8 Panigrahi, R., Panda, S., Behera, P. K., Sahu, S. K. & Rout, L. Recyclable bimetallic $CuMoO_4$ nanoparticles for C–N cross-coupling reaction under mild conditions. *New J. Chem.* **43**, 19274-19278 (2019).

9 Zhou, G. *et al.* A newly designed carbohydrate-derived alkylamine promotes Ullmann type C–N coupling catalyzed by copper in water. *Synlett.* **30**, 193-198 (2019).

10 Schulz, T. *et al.* Electronic effects of para-substitution on the melting points of TAAILs. *Chem Asian J.* **6**, 863-867 (2011).
